# Freezing stress response of wild and cultivated chickpeas

**DOI:** 10.3389/fpls.2023.1310459

**Published:** 2024-02-05

**Authors:** Shweta Kalve, Megan Alexandra House, Bunyamin Tar’an

**Affiliations:** Department of Plant Sciences, University of Saskatchewan, Saskatoon, SK, Canada

**Keywords:** cold, abiotic stress, wild chickpea, cultivated chickpea, RNA sequencing, QTL sequencing

## Abstract

Chickpea is an economically and nutritionally important grain legume globally, however, cold stress has adverse effects on its growth. In cold countries, like Canada where the growing season is short, having cold stress-tolerant varieties is crucial. Crop wild relatives of chickpea, especially *Cicer reticulatum*, can survive in suboptimal environments and are an important resource for crop improvement. In this study, we explored the performance of eleven *C. reticulatum* wild accessions and two chickpea cultivars, CDC Leader and CDC Consul, together with a cold sensitive check ILC533 under freezing stress. Freezing tolerance was scored based on a 1-9 scale. The wild relatives, particularly Kesen_075 and CudiA_152, had higher frost tolerance compared to the cultivars, which all died after frost treatment. We completed transcriptome analysis via mRNA sequencing to assess changes in gene expression in response to freezing stress and identified 6,184 differentially expressed genes (DEGs) in CDC Consul, and 7,842 DEGs in Kesen_075. GO (gene ontology) analysis of the DEGs revealed that those related to stress responses, endogenous and external stimuli responses, secondary metabolite processes, and photosynthesis were significantly over-represented in CDC Consul, while genes related to endogenous stimulus responses and photosynthesis were significantly over-represented in Kesen_075. These results are consistent with Kesen_075 being more tolerant to freezing stress than CDC Consul. Moreover, our data revealed that the expression of CBF pathway-related genes was impacted during freezing conditions in Kesen_075, and expression of these genes is believed to alleviate the damage caused by freezing stress. We identified genomic regions associated with tolerance to freezing stress in an F2 population derived from a cross between CDC Consul and Kesen_075 using QTL-seq analysis. Eight QTLs (P<0.05) on chromosomes Ca3, Ca4, Ca6, Ca7, Ca8, and two QTLs (P<0.01) on chromosomes Ca4 and Ca8, were associated with tolerance to freezing stress. Interestingly, 58 DEGs co-located within these QTLs. To our knowledge, this is the first study to explore the transcriptome and QTLs associated with freezing tolerance in wild relatives of chickpea under controlled conditions. Altogether, these findings provide comprehensive information that aids in understanding the molecular mechanism of chickpea adaptation to freezing stress and further provides functional candidate genes that can assist in breeding of freezing-stress tolerant varieties.

## Introduction

Cold stress is one of the most critical environmental stresses that greatly limits plant growth, survival, and productivity (Yadav, 2010; Zhu, 2016). It can be classified as chilling (<20°C) and freezing (<0°C) stress (Chinnusamy et al., 2007), with freezing temperatures having adverse effects on plant development. Short duration exposure to freezing temperatures, even just a few hours, can affect any stage of plant growth such as germination, early and/or late vegetative development, or reproduction (Devasirvatham et al., 2015; Kaloki et al., 2019; Li et al., 2022; Rastgoo et al., 2023). Moreover, freezing stress can influence physiological and biochemical processes in a manner that depends on the plant species, plant developmental stage, and length of the stress period (Jian et al., 2020).

Plants have various survival strategies by which they respond to and tolerate the cold stress. These can include changes in the structure, composition, and function of plasma membrane (Ding et al., 2019). After sensing the cold stress, cells undergo an increase in their membrane rigidity due to a reduction in the plasma membrane fluidity (Los and Murata, 2004). This increase in rigidity also increases electrolytic leakage from the cell and triggers the expression of cold-responsive genes, which acts as the primary signal for initiating a cold response (Dasgupta et al., 2020). Low temperatures, through membrane rigidification and/or other cellular changes, activate the MAPK signaling cascade and influx of cytoplasmic Ca^2+^ via mechano-sensitive Ca^2+^ channels (Chinnusamy et al., 2006; Chinnusamy et al., 2007; Ding et al., 2019). The increase of cytosolic calcium activates various downstream signaling pathways, mainly through calcium-dependent protein kinases (CDPKs; also called CPKs), Calcineurin B-like proteins (CBLs), and CBL-INTERACTING PROTEIN KINASES (CIPKs), which further activates the transcription of cold-responsive transcription factors (TFs) belonging to C-repeat binding factor (CBF)/Dehydration responsive element binding (DREB) family (Reddy et al., 2011; Ding et al., 2019). The DREB transcription factors directly bind to the promoters of cold-responsive (COR) genes and induce their expression, thereby enhancing freezing tolerance. COR refers to a class of genes regulated by cold stress such as *COLD REGULATED (COR), LOW TEMPERATURE INDUCED (LTI) and COLD INDUCIBLE (KIN)*. The DREB regulon genes play an important role in stabilizing membrane structure, activating reactive oxygen species (ROS) scavengers, and promoting the production of osmoprotectants to protect both the membrane and organelles from damage during cold stress (Guy, 1990; Cook et al., 2004; Hannah et al., 2005; Maruyama et al., 2009; Liu et al., 2018; Ding et al., 2019).

Chickpea (*C. arietinum* L.) is an important pulse legume cultivated and consumed worldwide (Varshney et al., 2013; Merga et al., 2019). Due to its richness in protein, fiber, and minerals it is also a crucial source of nutrition to millions of people globally (Jukanti et al., 2012; USDA, 2021). Chickpea is a cool season crop and has been suggested to perform optimally in temperatures of 21-26°C during the day and 18-21°C at night. Among abiotic stresses, low temperature is considered one of the main constraints affecting chickpea production (Rani et al, 2020). Freezing temperatures during the seedling and early vegetative stages of crop growth are considered a severe problem for winter or early spring sown chickpeas and are detrimental to chickpea yield (Kaloki et al., 2019; Khoshro and Lotfi, 2021). Therefore, development of freezing tolerant chickpea cultivars is needed to successfully prevent the damage that can occur following winter sowing and/or early spring frost.

The narrow genetic base of cultivars is a bottle neck for crop improvement; therefore, the use of crop wild relatives (CWR) is a promising approach to enhance genetic diversity of cultivated crops (Hajjar and Hodgkin, 2007; Zhang H. et al., 2019). However, crop wild relative taxa are frequently hindered in their effective utilization due to genetic compatibility issues (Kahraman et al., 2017), although, certain wild species like *C. reticulatum* are the closest genetically related crop wild relatives to the cultivated species *C. arietinum* and exhibit complete cross-compatibility. Various studies have used CWRs as sources to improve cold stress tolerance in many cultivated species (Koseki et al., 2010; Cruz et al., 2013; Coyne et al., 2020). Several studies have assessed the effects of chilling or freezing stress on chickpea in the field (Srinivasan et al., 1999; Heidarvand et al., 2011; Habibpour et al., 2012; Mir et al., 2021), but field studies are often associated with unpredictable severity and irregularity of cold stress (Maqbool et al., 2010). Therefore, studies under controlled conditions provide more precise information on the impact of cold stress on plants. Some studies have explored the response of chickpea to cold stress under controlled conditions (Zeitelhofer et al., 2022). Another study identified quantitative trait loci (QTLs) related to cold stress tolerance in chickpea using a population derived from ICC 4958×PI 489777, which was phenotyped for cold tolerance in the field over four field seasons and twice under controlled conditions (Mugabe et al., 2019). Using QTL mapping, the researchers identified three QTLs, each on LG1B, LG3, and LG8. Of these, one QTL was detected in a single environment while the other two QTLs were important in all six environments (Mugabe et al., 2019). Another recent study has reported a few cold responsive genes and proposed potential molecular mechanism related to cold stress tolerance in chickpea (Akbari et al., 2023). More specifically, the researchers found that photosynthesis was severely affected by cold stress.

In the current study, we investigated the performance of eleven *C. reticulatum* wild accessions and two chickpea cultivars, CDC Leader and CDC Consul under freezing stress. Moreover, we used Illumina sequencing data to investigate, and compare, responses of wild chickpea (*C. reticulatum L.*) and cultivated chickpea to freezing stress at an early vegetative stage. Using RNA sequencing, we identified differentially expressed genes (DEGs) that may impact freezing stress response in chickpea and characterized them using gene ontology (GO) enrichment analyses. Furthermore, using phenotypically extreme individuals from an F2 population derived from a cross between chickpea cultivar CDC Consul and a wild accession Kesen_075 we performed a QTL-seq analysis and identified QTLs associated with freezing stress resistance. Finally, we assembled a list of candidate freezing-stress response genes by identifying DEGs that co-localize with the QTLs and homologues of previously identified cold-response genes. These results provide insight into the potential mechanisms of freezing stress responses and can be used to compare freezing tolerance and sensitivity in chickpea. To the best of our knowledge, this study marks the pioneering investigation into both the transcriptome and QTLs linked to freezing tolerance in the wild counterparts of chickpeas under controlled environments. The results of this study, therefore, provide valuable resources to improve freezing tolerance in chickpea.

## Materials and methods

### Plant materials, experimental design, and cold treatment

*C. reticulatum* wild accessions (Kesen_075, CudiA_152, Bari3_106D, Savur_063, Rdsde_065, Sirna_060, CudiB_022C, Oyali_084, Cermi_075, Bari3_072C and Bari2_072), two chickpea cultivars (CDC Consul and CDC Leader) and a cold sensitive check (ILC533) were used in this study. Plants were grown in 4-inch styrofoam pots with Sunshine^®^ mix #4 (Sungro Horticulture, US) in the Phytotron facility at the University of Saskatchewan. Plants were grown under control conditions (day/night cycle of 14h-22°C/10h-16°C) for two weeks until plants reached the 5-6 node stage. After two weeks, one set of plants (with three replicates of each genotype) were moved to 4°C for one week for cold acclimation before the cold-stress treatment. Another set of plants (with two replicates of each genotype) were non-acclimatized and remained in control conditions until the cold-stress treatment. After the cold acclimation period, all acclimatized and non-acclimatized plants were exposed to -6°C for a total of 24 hours (h). After 3h, 6h, and then every 6h following (up to 24h), plants were moved to 4°C and then transferred to a chamber set to original control conditions to recover. During the recovery period, every week up to three weeks, freezing tolerance was scored based on a 1-9 rating scale, where 1 = no visible symptoms of damage and 9 = 100% plant killing (Singh et al., 1989). Moreover, iron and zinc concentrations in untreated seeds of Kesen_075 and CudiA_152 were measured as milligrams per kilogram (ppm) according to Diapari et al., 2014.

### RNA extraction

Aerial parts of the plant (whole shoot tissue) of cold tolerant wild accession Kesen_075 and cold sensitive cultivar CDC Consul were harvested for RNA extraction: (1) during control conditions, (2) after one week of cold acclimation, (3) following 3h, 6h, 12h and 24h of freezing treatment at -6°C, and (4) after one week of recovery under control conditions (samples were collected from 6h of cold treatment as cultivars treated with 12 and 24h of cold treatment died during recovery). Three replicates were used for each sample. Fresh tissue samples were immediately frozen in liquid nitrogen and stored at -80°C. Glass beads were added to individual samples and were ground using a Genogrinder. Total RNA of each individual sample was extracted and treated with DNase I, using a Qiagen RNeasy Plant mini kit (Qiagen, Ontario) following manufacturer’s instructions. Purified RNA quantity was determined by an optical density reading at 260 nm and the OD260/OD280 absorption ratio using NanoDrop 800 UV-vis spectrophotometer (Thermo Fisher Scientific, Inc. USA) and RNA integrity was checked on a 1.5% agarose gel using electrophoresis.

### Processing and mapping of Illumina 150 bp paired-end reads

Library preparation and mRNA-seq was performed by LGC Genomics GmbH (Berlin, Germany) using the Illumina NextSeq 500/550 v2 and NovaSeq 6000 platform. Sequencing generated 150 bp paired-end reads. LGC Genomics GmbH performed a quality assessment using the *FastQC* program (Andrews, 2010) and removed adapter remnants using the Illumina bcl2fastqv2.20 software (https://support.illumina.com/sequencing/sequencing_software/bcl2fastq-conversion-software/downloads.html). Using *STAR* (version 2.7.9a) (Dobin et al., 2013), we aligned clean, paired-end reads to gene sequences using the published v1.0 CDC Frontier reference genome (Ca_v1.0_kabuli_ref.fasta) and the corresponding annotation file (Ca_v1.0_kabuli_annotated_gene.gff3) (http://www.cicer.info/databases.php), which was converted to gtf format using the *GFF Utilities* function *gffread()* (Pertea and Pertea, 2020). We used default parameters within *STAR*, apart from the following: (1) –outFilterMismatchNmax set to ‘15’, and (2) –quantMode set to ‘GeneCounts’ so that the number of concordant read-pairs were counted for each gene.

### Identification of DEGs

Analysis of differential expression was performed in *Rstudio* (version 4.1.0) (R Core Team 2018; https://www.r-project.org/) using functions from the Bioconductor package *EdgeR* (Robinson et al., 2010). Genes with low read counts were filtered using the *filterByExpr()* function and remaining genes were used for tests of differential expression. The counts were normalized for library size using the TMM method and the function *calcNormFactors*(). Dispersion estimates were calculated based on shared information across genes (i.e. common dispersion) using the *estimateGLMCommonDisp()* function. We used the *glmLRT()* and *glmQLFTest()* functions to identify genes that are differentially expressed within genotypes and between cold acclimation and all other conditions, such as control, 3hr, 6hr, 12hr and 24hr of freezing stress, and also between control and recovery. Results were adjusted for multiple testing using a False Discovery Rate correction (Benjamini and Hochberg, 1995) and genes with FDR adjusted *p*-values of less than 0.05 and log_2_FC > 1 or log_2_FC < -1 was considered as significantly differentially expressed.

### Analysis of gene ontology enrichment

Enrichment of GO terms was tested using AgriGO 2.0 software (Tian et al., 2017). Enrichment tests were performed separately on genes that are up and downregulated between conditions (cold acclimation vs control; 3hr, 6hr, 12hr and 24hr of freezing stress vs cold acclimation; and recovery vs control) within each genotype. The reference list of GO terms is based on those for Arabidopsis homologues of chickpea genes, which were identified by Edwards (2016) (http://www.cicer.info/databases.php; *Kabuli* V1.0 raw data file). We used the TAIR 10 Arabidopsis GO terms for chickpea homologues (The Arabidopsis Information Resource (TAIR). Available at: https://www.arabidopsis.org/download/index-auto.jsp?dir=%2Fdownload_files%2FGO_and_PO_Annotations%2Fgene_Ontology_Annotations). GO term analysis in AgriGo 2.0 was run using the following parameters: a) hypergeometric test, b) complete GO, c) a minimum of 10 mapping entries, and d) Benjamini and Hochberg (Benjamini and Hochberg, 1995) FDR correction to adjust *p* values for multiple testing.

### Freezing stress screening and selection of F2s for resistant and susceptible bulks

A cross was made in the greenhouse between cold tolerant accession, Kesen_075, and cold sensitive cultivar, CDC Consul. A single F1 plant was selfed to generate the F2 population (n=197), which was used to identify the genomic regions associated with resistance to freezing stress. Plants were grown under control conditions (day/night cycle of 14h-22°C/10h-16°C) until the 3-4 node stage and leaf samples of all the F2s individuals and parents were collected for DNA extraction. After harvesting leaves, plants recovered for a week and were then moved to 4°C for one week for cold acclimation. After the cold acclimation period all the plants were treated for 24h at -6°C. Further, plants were moved to 4°C for 24hr and then transferred to a chamber with original control conditions to recover. During the recovery period, every week up to three weeks, freezing tolerance was scored based on a 1-9 rating scale (Singh et al., 1989). Selection of the F2s with the most extreme responses to freezing temperature (i.e., the most tolerant and the most sensitive) was based on the frequency distribution. The 17 most tolerant (plants with a cold tolerance score < 4) and 17 most sensitive (plants with a cold tolerance score>7.5) F2s were selected to form the tolerant and sensitive bulks.

### DNA extraction, library preparation and whole genome sequencing

Genomic DNA was isolated from the selected F2s individually using Qiagen DNeasy Plant Mini kit (Qiagen, Ontario) following manufacturer’s instructions. DNA quality was evaluated by running the samples on a 1% agarose gel and DNA was quantified using NanoDrop 800 UV-vis spectrophotometer (Thermo Fisher Scientific, Inc. USA). DNA concentration was adjusted to a final concentration of 100 ng/µl for each sample, and then 1 µg of total DNA from each F2 sample was used for bulking. Two DNA pools were generated by equally mixing DNA (one pool from DNA of the 17 most freezing-tolerant individuals and the other pool from DNA of the 17 most freezing-sensitive individuals). The pooled DNA samples and DNA samples from parental genotypes (CDC Consul and Kesen_075) were sequenced at the sequencing facility of LGC Genomics GmbH (Berlin, Germany) using the Illumina NextSeq 500/550 v2 sequencing system.

### QTL-seq analysis

To identify QTL, we used an established QTL-seq pipeline (Sugihara et al., 2022). This approach used the sequence data for the two bulked (or pooled) samples, described above, the reference sequence for CDC Frontier (http://www.cicer.info/databases.php), and default parameters for the QTL-seq pipeline.

### Statistical analysis

Standard deviation was calculated from three replicates of cold tolerance ratings for *C. reticulatum* wild accessions, chickpea cultivars and cold sensitive check. Statistical analyses to compare significant differences in Fe and Zn concentration between wild accessions, CudiA_152 and Kesen_075 were performed using t-tests (P ≤ 0.001).

## Results

### Comparison of responses to freezing temperatures in a chickpea wild accession and cultivar

**Figure 1:**
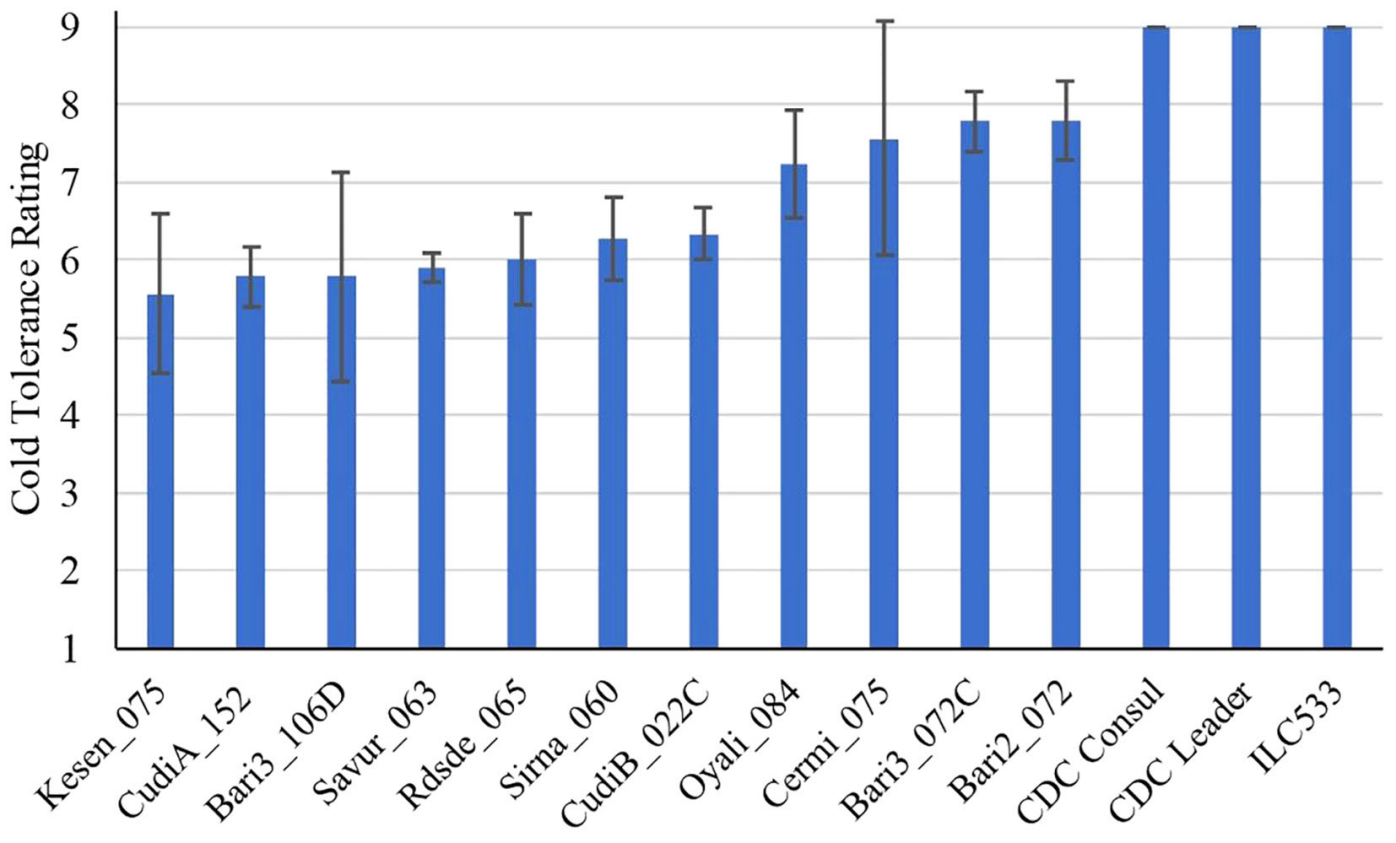
Cold tolerance ratings of *C. reticulatum* wild accessions, chickpea cultivars (CDC Consul and CDC Leader) and cold sensitive check (ILC533) after 2 weeks of recovery following 24 h of freezing stress at -6°C (n=3). Data are presented as mean ± SD.

To investigate and compare the responses of wild (*C. reticulatum* L.) and cultivated chickpeas (*C. arietinum* L.) to freezing temperatures, we examined the performance of 11 wild accessions, two cultivars, and a cold-sensitive check variety after two weeks of recovery following a 24h freezing treatment at -6°C. Our recovery data showed that four wild accessions (Kesen_075, CudiA_152, Bari3_106D, Savur_063) had cold tolerance ratings below 6, indicating they performed better than other wild accessions, which had an average rating of 7 (P<0.01), whereas chickpea cultivars did not survive the stress and died after the cold treatment with an average rating of 9 (P<0.001) (**Figure 1**).

**Figure 2:**
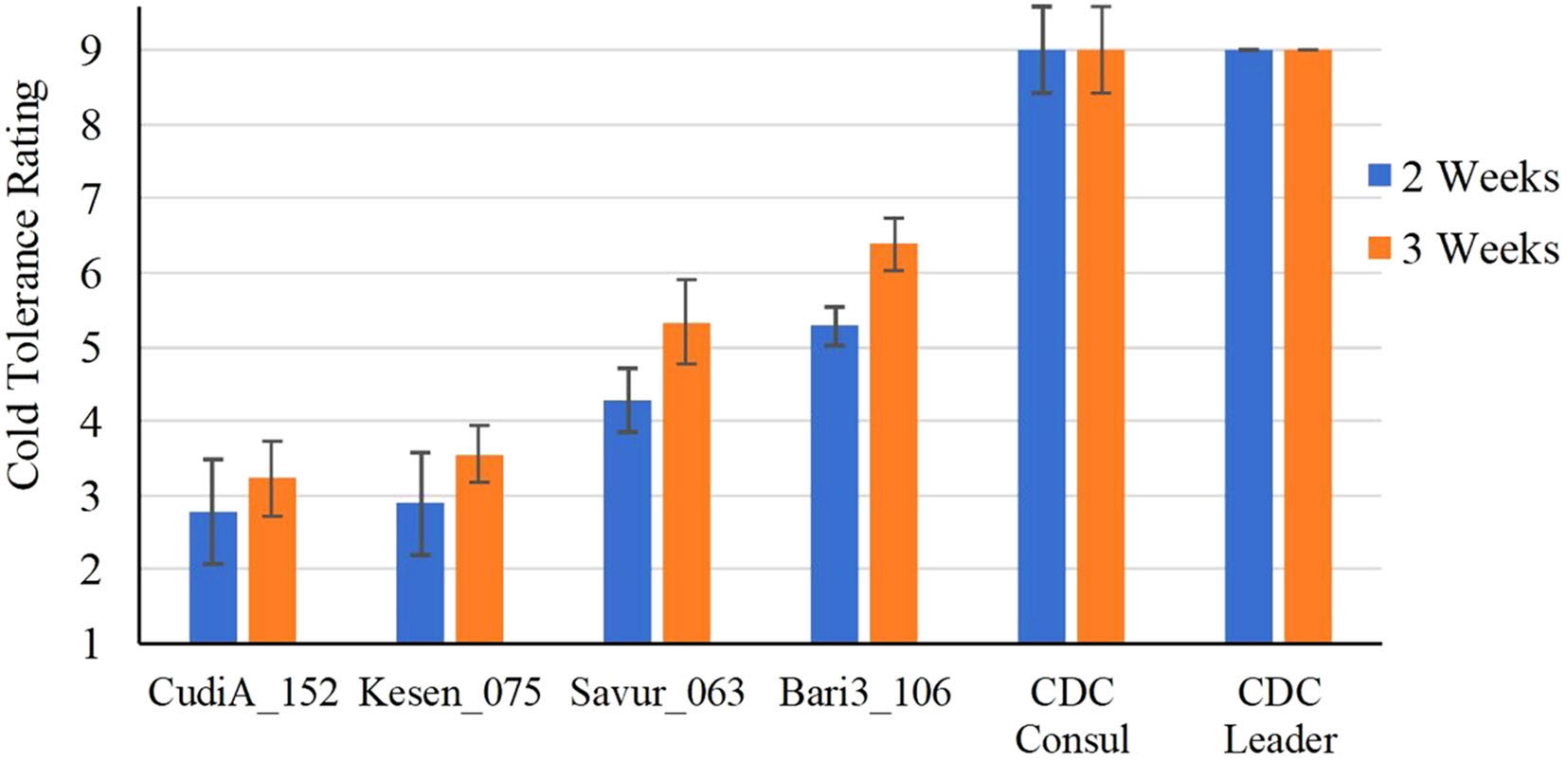
Cold tolerance ratings of selected *C. reticulatum* wild accessions and chickpea cultivars (CDC Consul and CDC Leader) after 2 and 3 weeks of recovery following 24 h of freezing stress at -6°C (n=3). Data are presented as mean ± SD.

**Figure 3:**
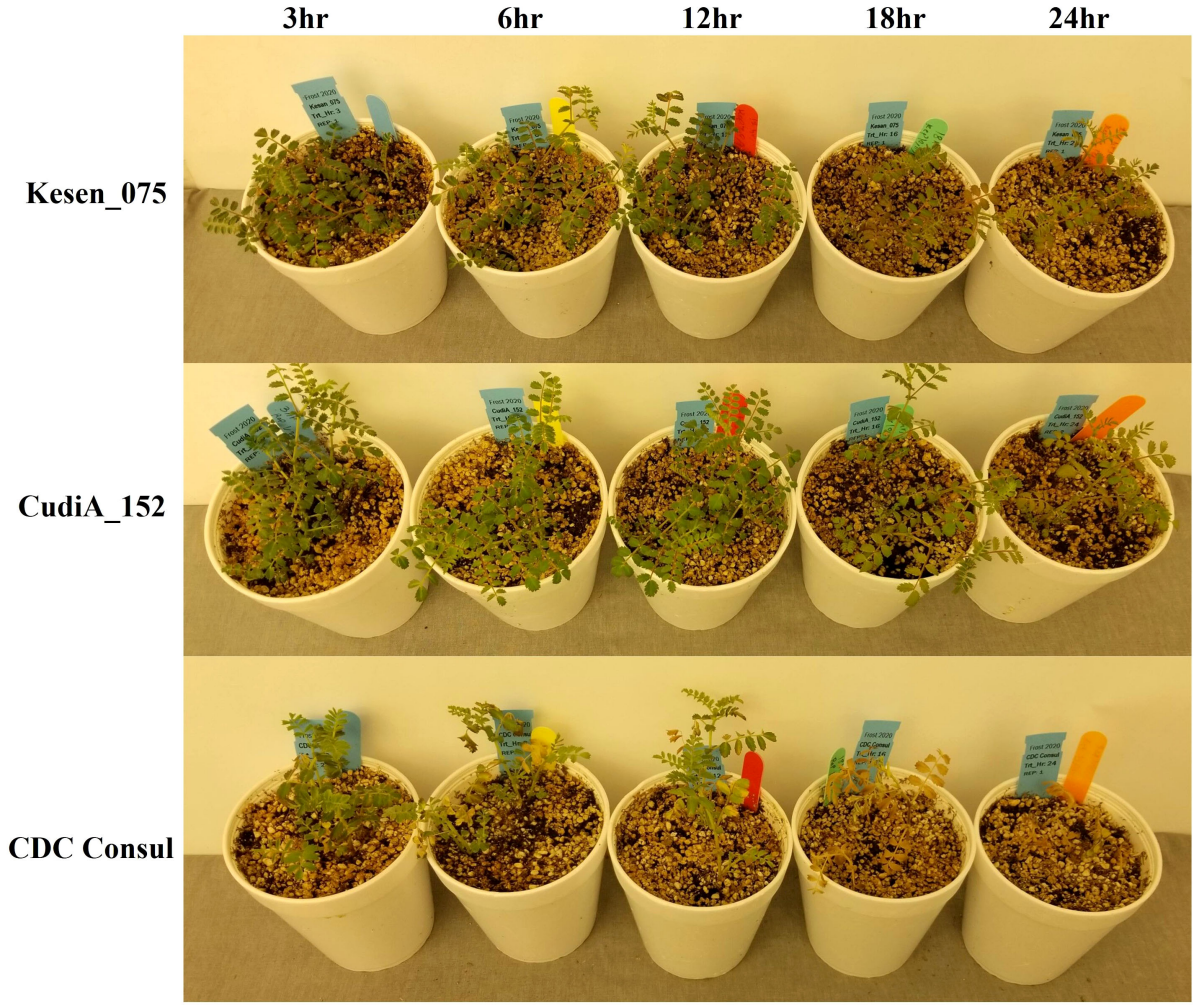
Wild accessions (Kesen_075 and CudiA_152) and chickpea cultivar (CDC Consul) one week after recovery following 3, 6, 12, 18 or 24 hours of freezing stress at -6°C.

The selected four most cold-tolerant wild accessions and two cultivars were further investigated in detail to determine the effect of a cold acclimation period on their response to freezing temperatures. Both sets of plants (acclimatized and non-acclimatized) were exposed to freezing temperatures and then returned to control conditions for recovery and assessed after two and three weeks. Interestingly, all non-acclimatized plants, including wild accessions, did not survive the freezing treatment and died after one week in recovery conditions (**Supplementary Figure 1**). Recovery data of acclimatized plants indicates that two wild accessions, CudiA_152 and Kesen_075, are the most cold tolerant (**Figure 2**). We observed development of new roots in the wild accessions following the freezing treatment. Moreover, anthocyanin pigmentation in the leaves and stems was observed in the wild accessions (**Figure 3**).

**Figure 4:**
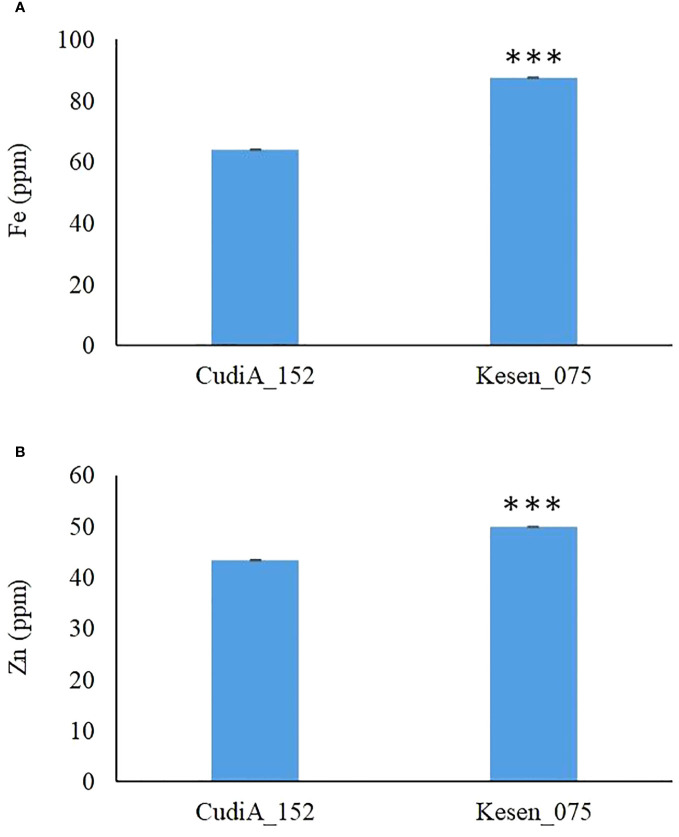
Iron **(A)** and zinc **(B)** concentrations in seeds of freezing tolerant *C. reticulatum* wild accessions, CudiA_152 and Kesen_075 (n=2). Data are presented as mean ± SD. *** indicates a significant difference at P ≤ 0.001.

Chickpea is a potential staple food to help reduce iron (Fe) and zinc (Zn) deficiencies in humans globally (Grewal et al., 2020). In addition to assessing cold-tolerance responses, we also measured the levels of important micronutrients Fe and Zn to determine if there is accumulation of these micronutrients in seeds of these two most cold tolerant wild accessions, CudiA_152 and Kesen_075. We found that the iron and zinc concentrations were significantly higher in the seeds of Kesen_075 than in CudiA_152 (P ≤ 0.001) (**Figure 4**). Therefore, we selected Kesen_075 for further investigation in our study.

### Gene expression changes observed in response to freezing temperatures in wild, and cultivated, chickpeas

**Figure 5:**
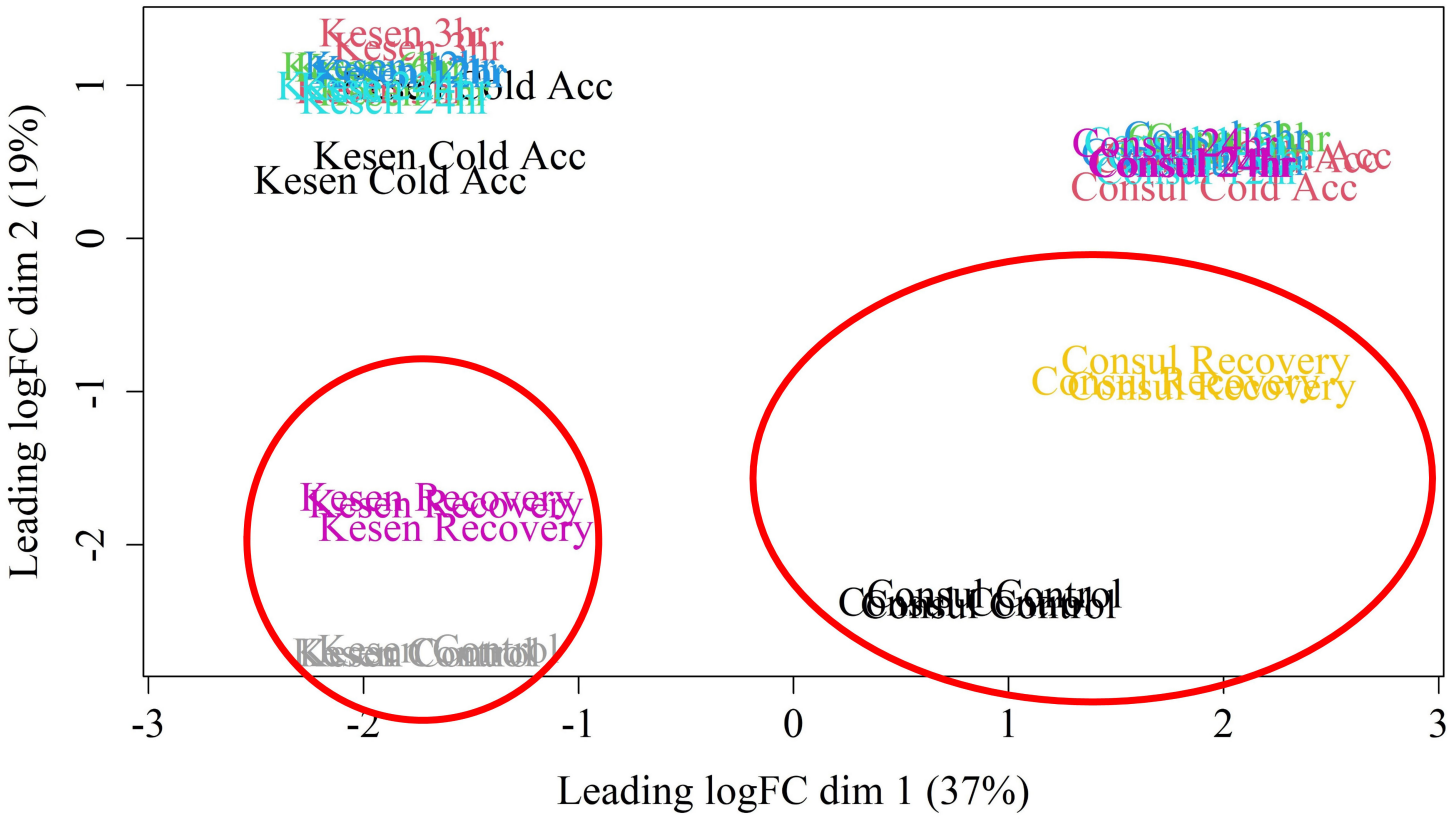
MDS plot of RNA-seq libraries. Red circles highlight the control and recovery samples from each genotype, which cluster closer together in cold tolerant accession Kesen_075 than in cold sensitive accession CDC Consul.

To understand the mechanism of cold stress tolerance in chickpea, we compared gene expression in whole aerial tissues of cold-tolerant wild accession Kesen_075 and cold-sensitive cultivar CDC Consul using an RNA-seq approach. RNA sequencing of samples from each treatment generated an average of 79 million paired-end reads per replicate, with approximately 91.6% uniquely mapping to the reference genome (**Supplementary Table 1**). Looking broadly at all samples, we used an MDS plot to identify potential outliers and found that all replicates within each sample clustered closely together. We also noted that samples within genotypes clustered closely together and were separated along the X axis, while samples within treatment groups also tended to cluster together along the Y axis (**Figure 5**).

**Figure 6:**
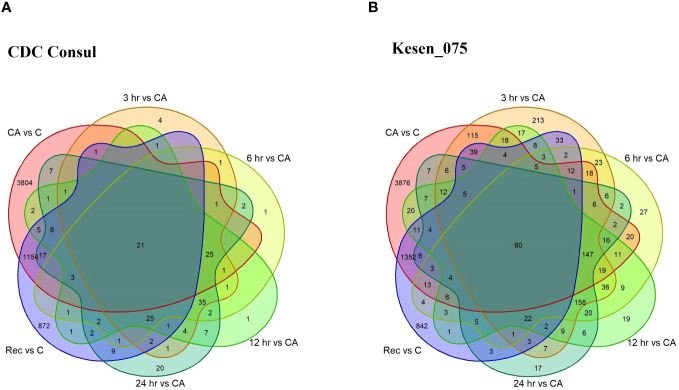
A venn diagram showing differentially expressed genes (DEGs) in cold acclimation (CA) vs control (C); 3hr, 6hr, 12hr and 24hr of freezing stress vs CA; and recovery (Rec) vs C in **(A)** CDC Consul and **(B)** Kesen_075.

**Figure 7:**
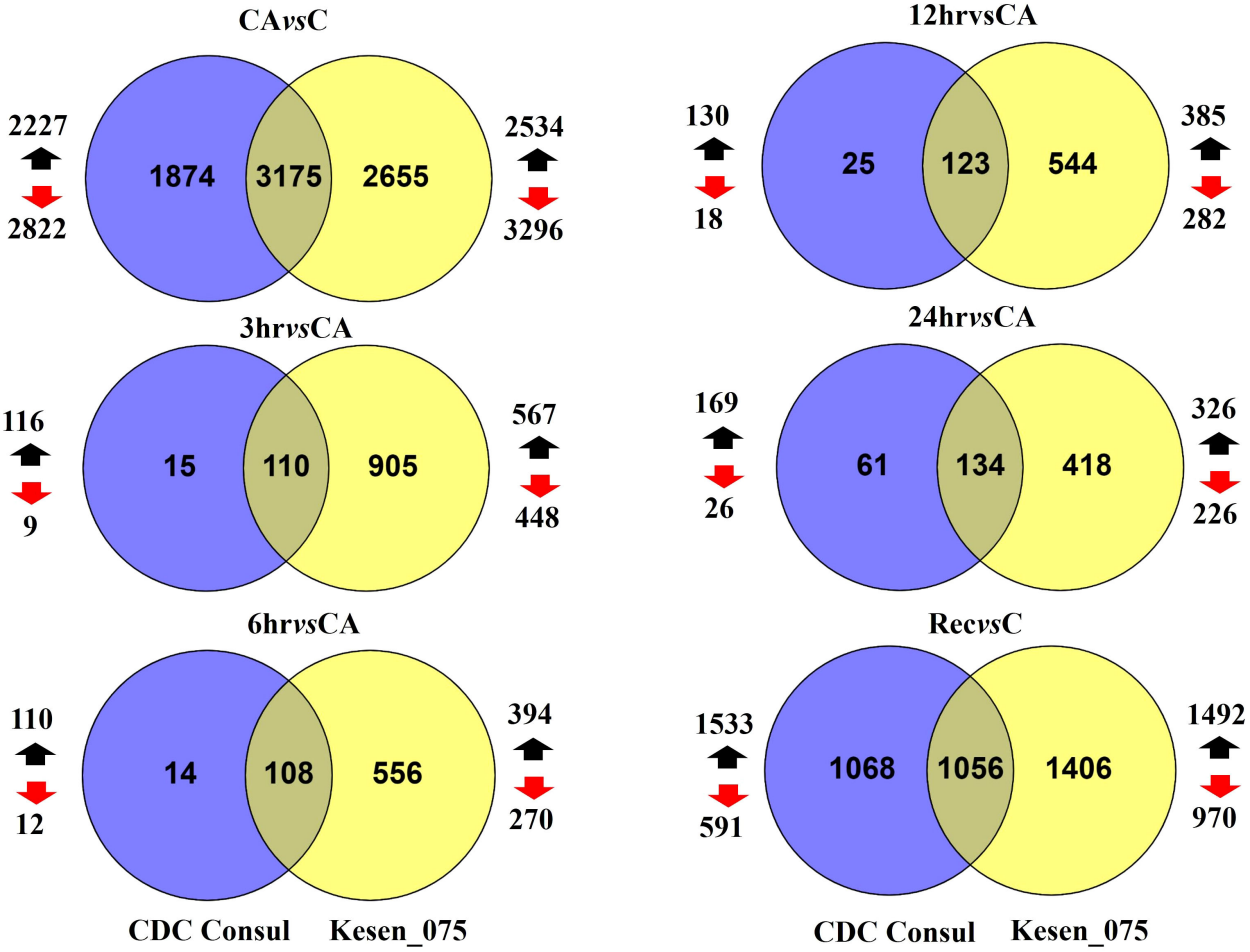
Venn diagrams showing the total number of DEGs in cold acclimation (CA) vs control (C); 3hr, 6hr, 12hr and 24hr of freezing stress vs CA; and recovery (Rec) vs C in CDC Consul (blue circles) and Kesen_075 (yellow circles). Genes that are differentially expressed in both genotypes are indicated where two circles overlap. The number of upregulated genes is shown by black arrows while the number of downregulated genes is shown by red arrows.

We identified genes that are differentially expressed between conditions [cold acclimation (CA) vs control (C); 3hr, 6hr, 12hr and 24hr of freezing stress vs cold acclimation; and recovery (Re) vs control] within each genotype (**Supplementary Tables 2A, B**). Hereafter, we will use term cold acclimation for CA*vs*C, 3hr for 3hr*vs*CA, 6hr for 6hr*vs*CA, 12hr for 12hr*vs*CA, 24hr for 24hr*vs*CA and recovery for Re*vs*C. Of 19,277 genes in the chickpea genome, 9,167 were differentially expressed in response to at least one treatment (FDR<0.05; log_2_FC > 1 or log_2_FC < -1, **Supplementary Table 2B**). Considering all pairwise comparisons made, we found that a total of 6,184 genes were significantly differentially expressed in CDC Consul, and 7,842 genes were differentially expressed in Kesen_075 (**Supplementary Table 2B**; **Figure 6**). Of these DEGs, 3,130 and 3,810 genes were significantly upregulated whereas 3,054 and 4,032 genes were significantly downregulated in CDC Consul and Kesen_075, respectively (**Supplementary Table 2B**; **Figure 7**).

### Functional annotation and enrichment analysis of DEGs

To understand the biological functions of the cold-induced DEGs, we conducted GO enrichment analyses. We first performed overall enrichment analyses on all DEGs within each genotype, regardless of the specific treatment comparison or the direction of expression change, and it revealed that genes related to stress responses, endogenous and external stimuli responses, secondary metabolite processes and photosynthesis were significantly over-represented in CDC Consul, while genes related to endogenous stimulus responses and photosynthesis were significantly over-represented in Kesen_075 (**Supplementary Figure 4**). We then tested for over-represented GO terms in the groups of genes either upregulated, or downregulated, between each pair of treatments within genotypes (**Supplementary Table 3**). We observed that ethylene, abscisic acid and salicylic acid mediated signaling, response to cold stress, anthocyanin-containing compound biosynthetic process, regulation of reactive oxygen species metabolic process and many others were the most commonly enriched GO terms among all the treatments in upregulated genes in both the genotypes (**Supplementary Tables 3A, C**). Within the set of downregulated genes, which largely consists of genes affected by cold acclimation and recovery, was the common enrichment in both genotypes of terms related to photosynthesis, stomatal complex development, cell cycle related, developmental process, metabolic process, among many others (**Supplementary Table 3B, D**).

As the majority of DEGs were detected during cold acclimation (**Figure 7**), we explored GO terms that were significantly enriched within up- and down-regulated genes in that period (**Supplementary Table 4**). It was observed that GO terms related to stress, hormone-mediated signaling pathway, endogenous stimulus, cell death, response to abiotic stimulus, defense, cell communication, negative regulation of response to stimulus etc. were among the GO terms commonly enriched within upregulated genes from both wild and cultivated genotypes (**Supplementary Table 4A**), while photosynthesis, cell cycle, developmental growth, pigment metabolic process etc. were among the commonly enriched GO terms within downregulated genes (**Supplementary Table 4B**) in both CDC Consul and Kesen_075 during cold acclimation.

### Effects of freezing stress on the ICE-CBF-COR pathway

**Figure 8:**
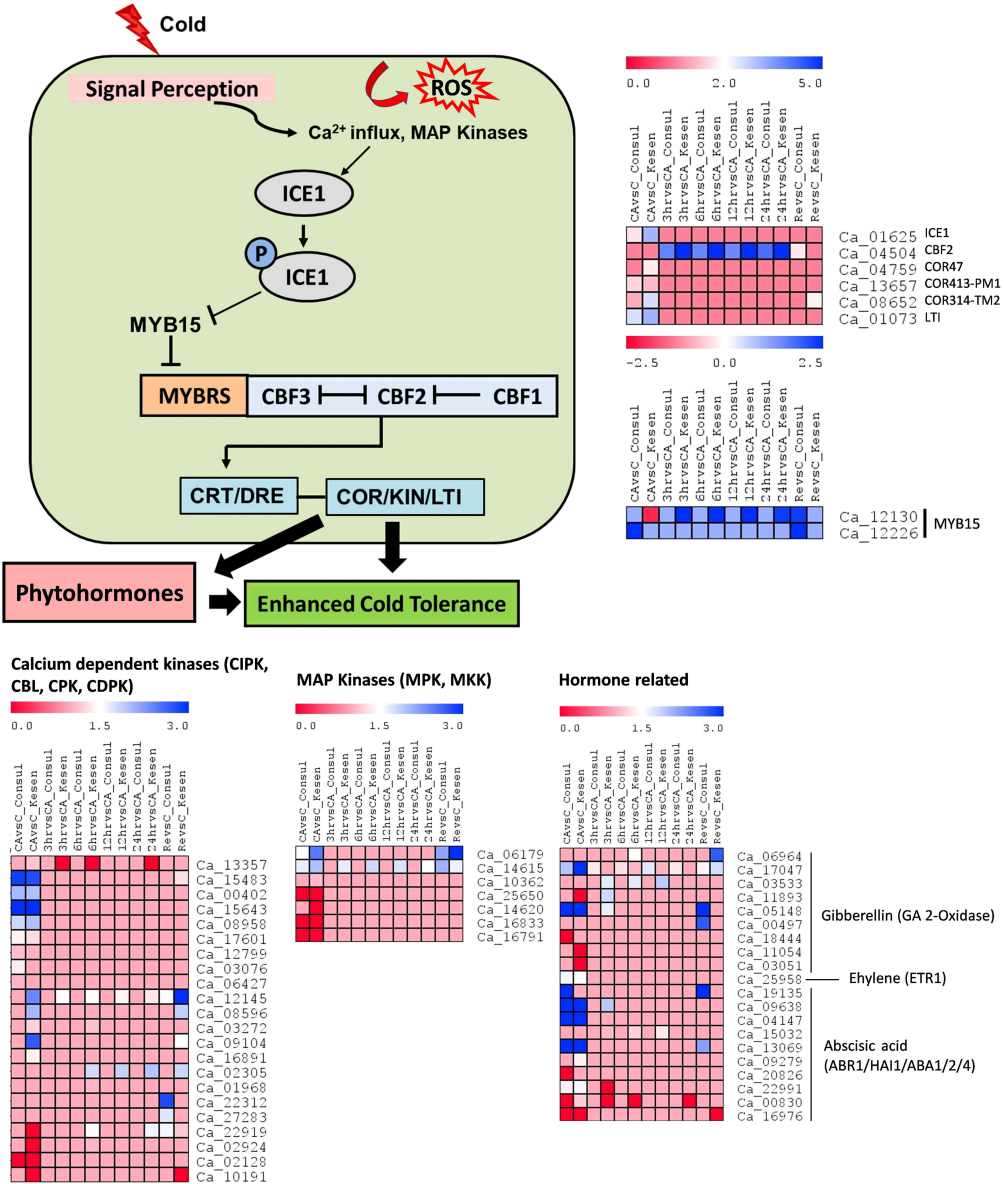
Schematic illustration of regulatory networks involved in low temperature responses: The onset of low temperature stress induces calcium influx, activating protein kinases. These kinases then initiate *ICE1* activation. *ICE1*, when activated, suppresses *MYB15* while inducing the expression of *CBFs*, which in turn govern the regulation of *COR* genes. ROS (Reactive oxygen species), *ICE1* (Inducer of CBF expression 1), *CBF* (C-repeat binding factor), *CRT* (C-repeat elements*), DRE (*Dehydration-responsive elements*), COR* (Cold-responsive genes), *KIN* (Cold-induced genes), *LTI* (Low temperature induced genes). The heatmap shows log2 fold changes in expression of CBF-mediated upstream and downstream genes in cold sensitive genotype CDC Consul and cold tolerant genotype Kesen_075 between treatments. Blue and red colours represent up- and down-regulation of genes, respectively.

Cold acclimation encompasses an array of physiological and biochemical adjustments. As most of the DEGs in our study occurred in response to cold acclimation, we explored an important and well-studied cold response pathway i.e., the ICE-CBF-COR signaling pathway. To understand the regulation of the ICE-CBF-COR pathway in detail, we examined the expression of the key regulators in CDC Consul and Kesen_075 (**Figure 8**). Specifically, we assessed expression changes resulting from the following sets of treatment comparisons: 1) control vs cold acclimation; cold acclimation vs (2) 3h, (3) 6h, (4) 12h and (5) 24h of cold stress; and (6) control vs recovery.

We found that the expression of most of the calcium dependent kinases and MAP kinases was higher in cold acclimation and recovery, relative to control conditions, in Kesen_075, whereas most of the members of these families were downregulated in CDC Consul (**Figure 8**). For example, one of the key genes in the ICE-CBF-COR pathway, *ICE1 (inducer of CBF expression)*, is upregulated in Kesen_075 under cold acclimation while in CDC Consul it is downregulated. Moreover, a negative regulator, *MYB15*, is downregulated in Kesen_075 under cold acclimation while it is upregulated in CDC Consul. Another important regulator *CBF2* and other downstream positive regulators such as *COR47*, *COR314* and *LTI* were upregulated in Kesen_075 but downregulated in CDC Consul, whereas the pattern of expression of *COR413* was similar in both the genotypes (**Figure 8**). Plant responses to cold stress are also regulated by various plant hormones, either by CBF-dependent or -independent mechanisms. In our study, 5 out of 9 genes encoding members of the GA-2-oxidase (GA2oxs) family, which consists of negative regulators of gibberellic acid and plant growth, were upregulated in Kesen_075, while only 2 members of this family were upregulated in CDC Consul (**Figure 8**). Expression of *ETR1 (Ethylene receptor 1)* was high during cold acclimation in both genotypes, and one member out of 3 of *ABR1 (ABA REPRESSOR1)* was upregulated while two members of *ABA2 (ABA DEFICIENT 2)* were downregulated in CDC Consul (**Figure 8**).

**Figure 9:**
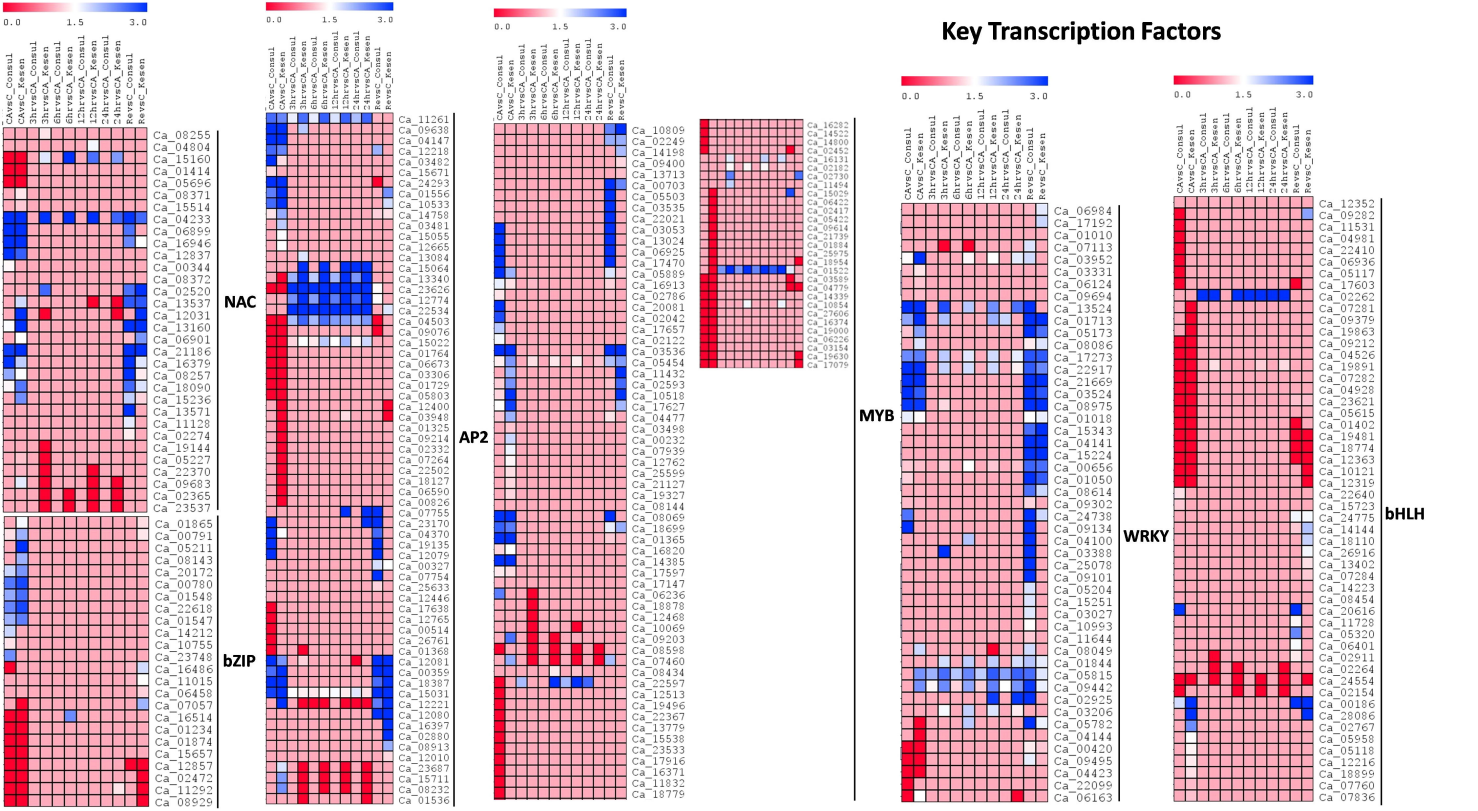
A heatmap showing the log2 fold changes in expression of genes encoding key transcription factors in cold sensitive genotype CDC Consul and cold tolerant genotype Kesen_075 between various treatments. Blue and red colours represent up- and down-regulation of genes, respectively.

We further analyzed the expression of key transcription factor genes [*NAC* (NAM, ATAF1/2, and CUC2), *bZIP* (basic leucine zipper), *AP2/ERF* (APETALA2/Ethylene Responsive Factor), *MYB* (v-Myb myeloblastosis viral oncogene homolog), *WRKY* (named because of the WRKYGQK heptapeptide at the N-terminal end) and *bHLH* (basic helix-loop-helix)], which have been reported to be involved in freezing stress (**Figure 9**). We identified a total of 32 *NAC*, 24 *bZIP*, 65 *AP2/ERF*, 90 *MYB*, 49 *WRKY* and 52 *bHLH* members amongst the DEGs in CDC Consul and Kesen_075. Comparison of the expression patterns of genes encoding these TFs between the two genotypes showed that the expression of a few members of some of the transcription factor gene families, such as *NAC*, *AP2*, *MYB* and *bHLH*, were high in Kesen_075 during cold acclimation, freezing treatment and recovery while a few members of these TF gene families were downregulated in CDC Consul mainly during freezing treatment (**Figure 9**). Expression of *WRKY* was increased in Kesen_075 during the freezing treatment while *bZIP* had similar expression patterns in both the genotypes (**Figure 9**).

**Figure 10:**
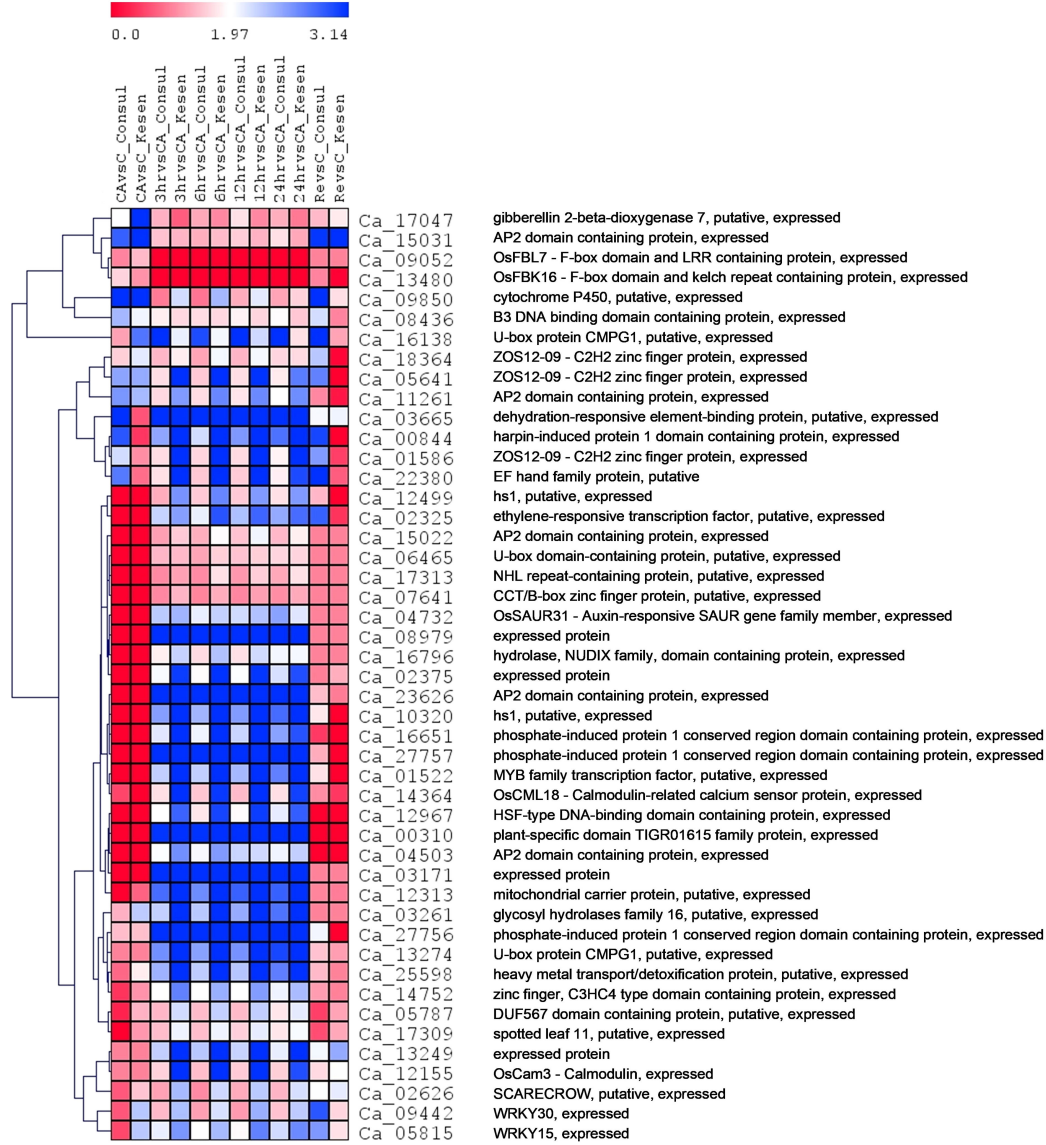
A heatmap showing the log2 fold changes in expression patterns of common DEGs (expressed between at least ten pairs of treatments across all twelve possible pairs when both genotypes are considered) between cold tolerant genotype Kesen_ 075 and cold sensitive genotype CDC Consul. Blue and red colours represent up- and down-regulation of genes, respectively. The vertical distances on each branch of the dendrogram represent the degree of similarity between gene expression profiles of all treatments in both the genotypes.

Moreover, 47 genes were significantly differentially expressed (up- or down-regulated) between at least ten pairs of treatments across all 12 possible pairs within Kesen_075 and CDC Consul (**Figure 10**). Among these genes were those encoding important transcription factors such as AP2/ERF/MYB/WRKY, U-box protein CMPG1 that has a role in plant defense, ZOS12-09 C2H2 zinc finger protein, GA2ox, gene coding EF hand protein, SCARECROW (SCR), which had higher expression in Kesen _075 while their expression was lower in CDC Consul (**Figure 10**).

### Effects of freezing stress on additional biological processes

**Figure 11:**
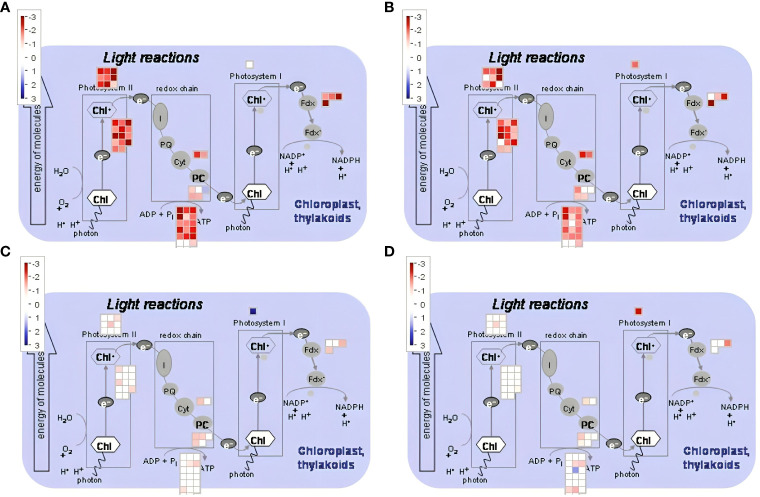
MapMan analysis (Thimm et al., 2004) was used to visually analyze genes involved in photosynthesis in response to cold acclimation and recovery treatments. Specific up- and down-regulated genes are indicated (blue and red squares, respectively) in **(A, B)** cold acclimation vs control and **(C, D)** recovery vs control in **(A, C)** chickpea cultivar CDC Consul and **(B, D)** wild accession Kesen_075.

**Figure 12:**
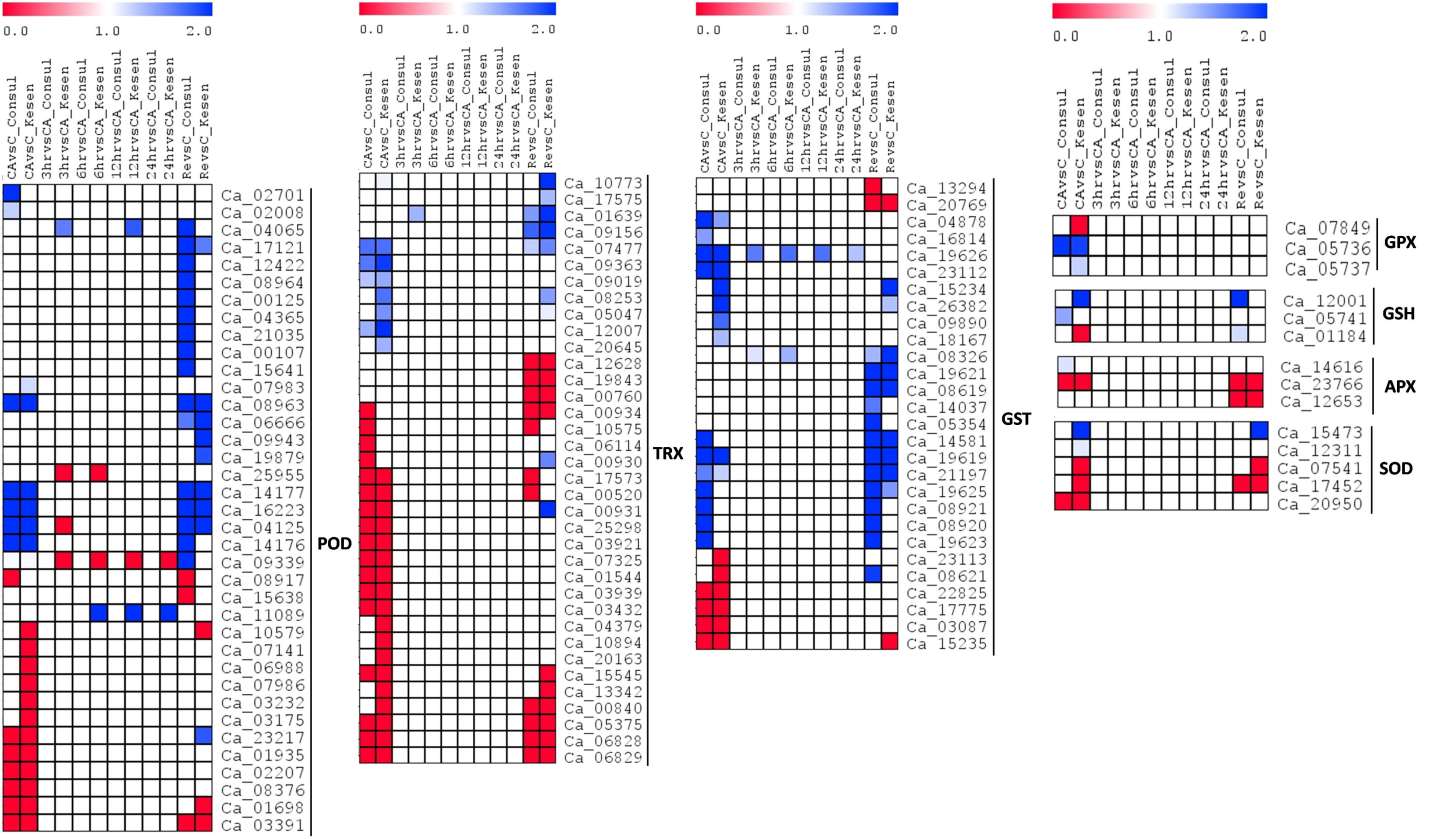
A heatmap showing the log2 fold changes of DEGs involved in ROS signaling in cold tolerant genotype Kesen_075 and cold sensitive genotype CDC Consul between treatments. Blue and red colours represent up- and down-regulation of genes, respectively. POD, peroxidase; TRX, thioredoxin; GST, glutathione S-transferase; GPX, glutathione peroxidase; GSH, glutathione; APX, ascorbate peroxidase; SOD, superoxide dismutase.

**Figure 13:**
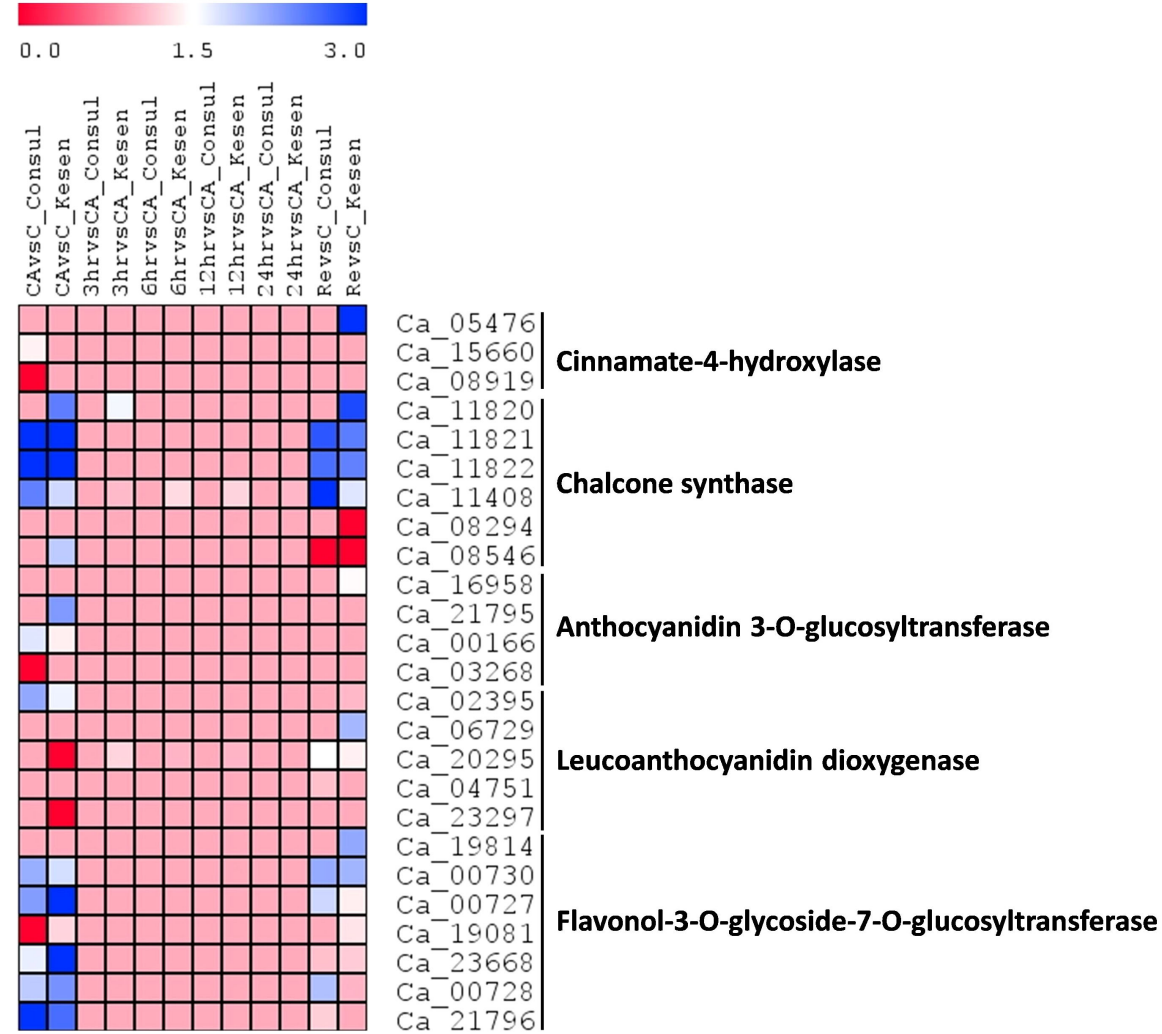
A heatmap showing the log2 fold changes of DEGs involved in anthocyanin biosynthesis in cold tolerant genotype Kesen_075 and cold sensitive genotype CDC Consul between treatments. Blue and red colours represent up- and down-regulation of genes, respectively.

We have found that in addition to genes in the ICE-CBF-COR pathway, there are others related to photosynthesis, oxidative stress responses, and metabolism of anthocyanin that had altered transcript abundance in both genotypes in response to freezing temperatures (**Figures 11**–**13**). GO term enrichment analysis showed that genes related to photosynthesis were significantly downregulated by freezing temperatures in both CDC Consul and Kesen_075 (**Supplementary Figure 4**, **Supplementary Table 4B**). We found that genes encoding elements of Photosystem II (PSII) were repressed in CDC Consul and Kesen_075 during cold acclimation while expression of only a single gene (*Ca_27778*) related to PSI was significantly downregulated in Kesen_075 (**Figure 11**). Moreover, during recovery, expression of genes related to PSII either remained stable or were downregulated in CDC Consul or Kesen_075, whereas one gene (*Ca_27778*) related to PSI was upregulated in CDC Consul and downregulated in Kesen_075 (**Figure 11**).

We further explored expression of genes related to oxidative stress responses (i.e., ROS scavenging and anthocyanin production) in tolerant and sensitive genotypes. Expression of 115 ROS scavenging genes was significantly affected in our study (**Figure 12**). Notably, 16 members out of 37 of the peroxidase (POD) family were upregulated in CDC Consul while 9 were upregulated in Kesen_075 during recovery. Additionally, two members of POD (*Ca_04065* and *Ca_11089*) were upregulated in Kesen_075 during freezing treatments (i.e., following 3, 6, 12 and 24h of freezing). Genes encoding members of the thioredoxin (TRX) and Glutathione S-transferase (GST) families were mostly differentially expressed during cold acclimation and recovery in both genotypes (**Figure 12**). Specifically, 8 genes encoding TRX had increased expression in Kesen_075 following recovery while two genes encoding of GST (*Ca_19626* and *Ca_08326*) were upregulated during all freezing treatments. Interestingly, genes encoding glutathione peroxidase (GPX), glutathione (GSH), ascorbate peroxidase (APX) and superoxide dismutase (SOD) were either up- or down-regulated during cold acclimation and recovery whereas no significant change was found during the freezing treatments in either genotype (**Figure 12**). One member (*Ca_07849*) of *GPX* was downregulated while another member (*Ca_5737*) was upregulated in Kesen_075 during cold acclimation and no change in expression was found in CDC Consul. On the other hand, *Ca_05736* had similar expression patterns in both the genotypes. Curiously, *Ca_12001*, a member of *GSH*, was upregulated in Kesen_075 during cold acclimation while in CDC Consul its upregulated during recovery. Moreover, *Ca_05741* was upregulated only in CDC Consul during cold acclimation and *Ca_01184* is downregulated in Kesen_075 during cold acclimation whereas was upregulated in CDC Consul during recovery. Remarkably, members of *APX* excluding *Ca_14616* had similar expression patterns in both the genotypes while one member of *SOD* (*Ca_15473*) was upregulated during both cold acclimation and recovery in Kesen_075 only (**Figure 12**).

Moreover, we observed anthocyanin pigmentation in some of the wild progenitors, including Kesen_075 during the freezing treatments, which may be involved in stress tolerance (**Figure 3**). Therefore, we investigated the expression of anthocyanin biosynthesis genes (**Figure 13**). We found that the expression of *Ca_05476* [*cinnamate-4-hydroxylase* (*C4H*)] was higher in Kesen_075 during recovery while there was no significant change in expression in CDC Consul. Both genotypes showed similar expression patterns of *chalcone synthase* (*CHS*) where 3 members were upregulated and one member (*Ca_08546*) was downregulated. Interestingly, only a single member had increased expression during cold acclimation and recovery specifically in Kesen_075. One member of *anthocyanidin 3-O-glucosyltransferase* was upregulated in Kesen_075 whereas one member was upregulated and other was downregulated in CDC Consul during cold acclimation. One member of *leucoanthocyanidin dioxygenase* was upregulated in CDC Consul while two members were downregulated in Kesen_075 in the duration of cold acclimation. Remarkably, major changes in gene expression of *flavonol-3-O-glycoside-7-O-glucosyltransferase* were found during cold acclimation where most members were upregulated in Kesen_075 while two members were up- and one was down-regulated in CDC Consul (**Figure 13**).

### The response of F2 population to freezing stress and development of bulk segregants

**Figure 14:**
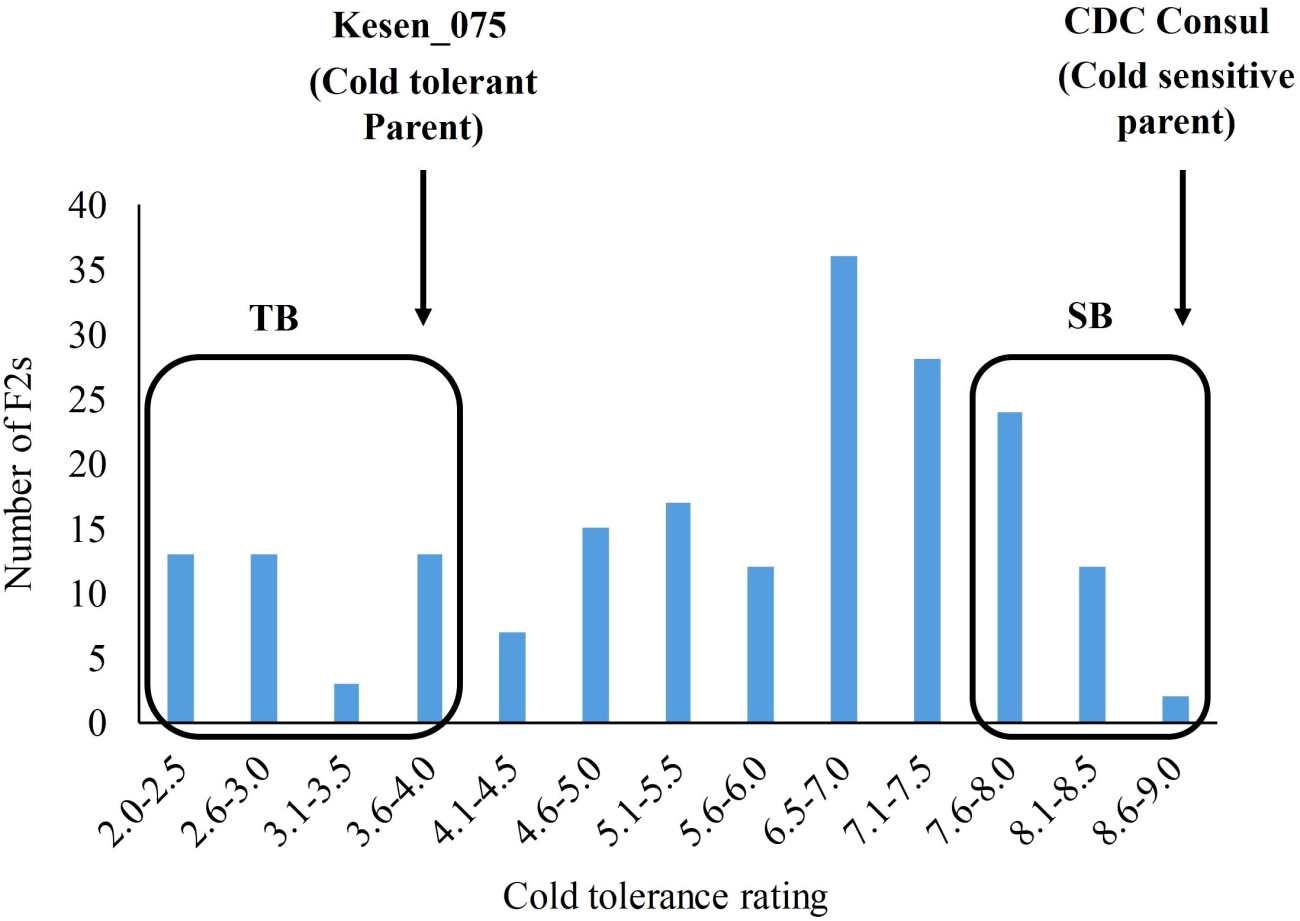
Frequency distribution of cold tolerance in an F2 population. Scores are based on a 1-9 scale, where 1 = no visible symptoms of damage and 9 = 100% plant killing (Singh et al., 1989). Arrows show the mean score of the tolerant (Kesen_075) and the sensitive (CDC Consul) parents. Based on the cold tolerance, 17 F2s exhibiting extreme cold tolerance ratings on both ends of the scale were selected to construct tolerant bulk (TB) and sensitive bulk (SB).

An F2 population (n=197) was developed from a cross between cold tolerant wild accession Kesen_075 and the cold sensitive cultivar CDC Consul. The cold tolerance scores among the F2s ranged from 2 to 9 (**Figure 14**; **Supplementary Figure 5**). Mean cold tolerance ratings between the parents differed significantly, with Kesen_075 having an average rating of 4 (ranging from 3-5) and CDC Consul consistently rating 9 (P<0.001). Based on the cold tolerance scores of the F2s, 17 individuals with the lowest and highest tolerance scores were selected and pooled as tolerant (TB) and sensitive (SB) bulks, respectively (**Figure 14**). The average cold tolerance score of the TB bulk was 3.0 (ranged from 2.0 to 4.0), while the average tolerance score of the SB bulk was 8.5 (ranged from 8.0 to 9.0). The frequency distribution of cold tolerance scores in F2 population followed a normal distribution pattern suggesting that the resistance to cold tolerance is polygenic and is likely controlled by multiple QTL (**Figure 14**).

### QTL-seq analysis

**Figure 15:**
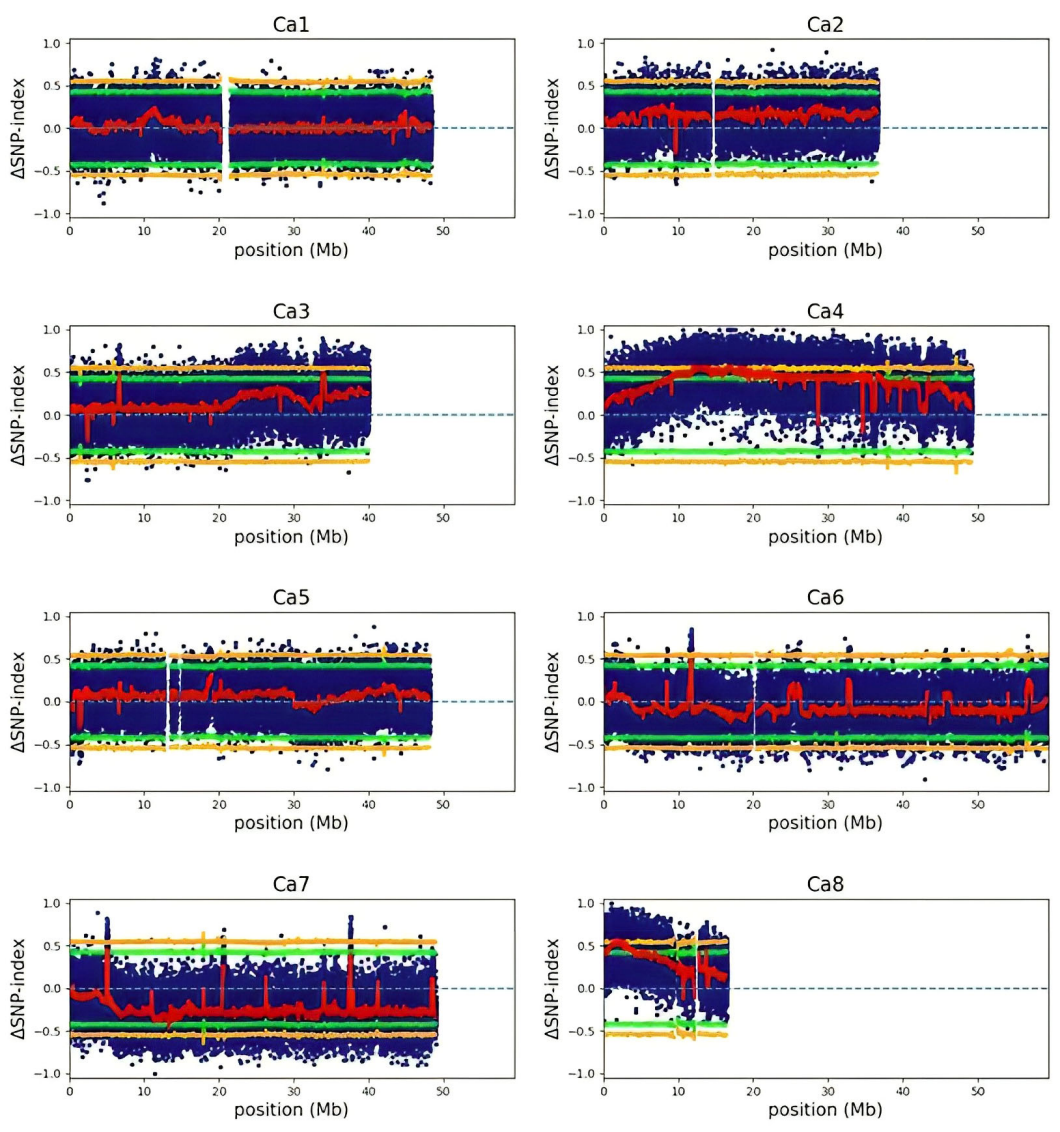
ΔSNP index plots of each chromosome generated by QTL-seq analysis. Blue dots represent individual variants. The red line indicates the mean SNP-index. Green and orange colours represent statistical confidence intervals (P<0.05 and P<0.01, respectively).

We used an established QTL-seq approach (Sugihara et al., 2022) to detect meaningful regions of the genome (QTL) that may help explain differences in the responses to freezing temperatures between CDC Consul and Kesen_075. We detected ten cold-tolerance QTLs with a ΔSNP index that surpassed a significance threshold of P<0.05, and these ten QTL had an average length of 4.59 Mb (**Figure 15**; **Supplementary Table 5**). We further investigated the ten QTL and identified 2681 genes within their borders (**Supplementary Table 5**). Among these are 58 that are both differentially expressed and have previously been reported to be involved in cold stress tolerance mechanisms (Shi et al., 2015; Shi et al., 2015; Zhu, 2016; Abdullah et al., 2022; Akbari et al., 2023; Raza et al., 2023) (**Supplementary Table 6**). Few of these genes were either up- or down-regulated in both the genotypes whereas some genes were significantly differentially expressed only in one genotype (**Supplementary Table 6**). Interestingly, genes encoding key regulators of cold tolerance mechanisms such as CBF2, key transcription factors such as AP2/MYB/WRKY/bHLH/NAC/bZIP, GA20ox (GA-20-oxidase), GA2ox, peroxidases, protein kinases were within QTLs and were also significantly differentially expressed (**Supplementary Table 6**).

## Discussion

Cold represents one of the primary environmental stresses that exerts a substantial influence on crop production. It hampers the growth, yield, and overall quality of various crop species (Yadav, 2010; Zhu, 2016; Kaloki et al., 2019). Plants, being immobile organisms, have developed diverse physiological, biochemical, and molecular mechanisms to counteract the effects of cold stress (Akbari et al., 2023; Rastgoo et al., 2023). These mechanisms are finely tuned by a complex network of transcription factors and proteins, all working together to enhance a plant’s ability to withstand cold conditions. In recent times, notable progress has been made in deciphering the mechanisms of perception and transmission of cold signaling in plants. Nonetheless, our understating of the molecular mechanisms triggered by low temperatures in chickpea remains incomplete. Therefore, our findings in this study yield valuable information regarding how cold-tolerant wild (*C. reticulatum*) chickpea and cold-sensitive cultivated (*C. arietinum*) chickpea genotypes respond to cold stress.

CWRs typically exhibit a greater degree of genetic and phenotypic diversity compared to their domesticated counterparts (Singh et al., 2008; Zhang H. et al., 2019). This diversity represents a valuable genetic resource for breeding programs. Numerous recent studies have harnessed CWRs as a valuable resource for enhancing the cold stress resilience of numerous cultivated plant species (Koseki et al., 2010; Cruz et al., 2013; Coyne et al., 2020). Moreover, it has been documented that wild relatives serve as a significant genetic resource for enhancing resistance to biotic and abiotic stresses in chickpea (Sharma et al, 2006; Singh et al., 2008; Coyne et al., 2020; Bakir et al., 2021; Kalve et al., 2022). *C. reticulatum* is situated within the primary gene pool of chickpea and is notable for its ability to readily crossbreed with domesticated chickpea, resulting in fertile offspring primarily due to the successful chromosome pairing observed during such crossings (Bakir et al., 2021; Kalve et al., 2022). Therefore, in our study we have explored the response of both wild and cultivated chickpea to freezing stress. In agreement with a previous study, we have also found that the chickpea wild relatives are more tolerant to cold stress (Mir et al., 2021). Our results revealed that the wild accessions, Kesen_075 and CudiA_152, are most cold tolerant in comparison to other wild accessions, whereas the chickpea cultivars, CDC Leader and CDC Consul, could not withstand the stress and succumbed after the cold treatment. Chickpeas have the potential to serve as a staple food that can contribute to reducing iron and zinc deficiencies in the global human population (Grewal et al., 2020) and of the two most cold tolerant wild accessions we examined, Kesen_075 stood out with significantly higher concentrations of both Fe and Zn in its seeds, holding promise for addressing micronutrient deficiencies in human diets.

To delve deeper into the molecular mechanisms underpinning cold stress tolerance, we conducted a transcriptome analysis comparing the cold-tolerant wild accession Kesen_075 with the cold-sensitive cultivar CDC Consul. Interestingly, it was observed in the MDS plot that the recovery and control samples cluster more closely together in Kesen_075 than in CDC Consul. This may indicate that following cold treatment, the transcriptome of Kesen_075 returns closer to ‘normal’, or pre-stress conditions, than in CDC Consul, which may suffer residual changes in expression. Differential gene expression analysis revealed the intricate interplay of gene regulation during cold stress. A substantial number of genes (9,167) exhibited differential expression in response to at least one treatment, underscoring the complexity of the response. Remarkably, most changes in transcript abundance occurred in response to cold acclimation, whereas fewer genes were affected by the freezing temperature treatments. Moreover, during the recovery period, plants partially restore transcript abundance compared to the control conditions.

In-depth annotation and enrichment analysis of the differentially expressed genes unveiled key insights into the biological processes that were affected by the various treatments. Interestingly, overall, genes related to stress responses, stimuli responses, secondary metabolite processes, and photosynthesis were significantly over-represented in CDC Consul, while Kesen_075 exhibited enrichment in genes linked to endogenous stimulus responses and photosynthesis. Similar responses to cold stress were observed in a recent study in chickpea where cold tolerant and sensitive genotypes were studied at 4°C under control conditions (Akbari et al., 2023). They found that the secondary metabolite pathway and photosynthesis were affected by the cold treatment (Akbari et al., 2023). Photosynthesis serves as the primary mechanism for harnessing light energy to produce carbohydrates and is disrupted during exposure to lower temperatures in chickpea (Zeitelhofer et al., 2022; Akbari et al., 2023). Moreover, other studies on different species also found a similar response where photosynthesis was affected. In the study conducted by Hajihashemi et al. (2018), it was observed that cold stress at 5°C resulted in PS II photoinhibition in nine cultivars of *Stevia rebaudiana*. Another study by Xu et al. (2022) also shows that cold treatment inhibits photosynthesis in *Camellia weiningensis* and *C. oleifera*. In our study, we have also found that genes related to photosynthesis, specifically those related to Photosystems I and II, were downregulated during cold acclimation in both tolerant and sensitive genotypes. Cold-induced photosynthetic inhibition may occur for several reasons, including reduced chlorophyll synthesis, inadequate chloroplast development, decreased efficiency of the photosynthetic machinery, restricted carbohydrate transport, limited stomatal conductance, suppressed Rubisco activity during carbon assimilation, disrupted electron transport chain, and reduced energy reserves (Bota et al., 2004; Hussain et al., 2018).

Investigation of transcriptional alterations in plants during cold acclimation is crucial for understanding the underlying molecular mechanisms involved in cold stress responses. There are many cold responsive genes and gene regulated networks that have been identified (Chinnusamy et al., 2007; Shi et al., 2015; Ding et al., 2019). Among these, the best understood is the ICE-CBF-COR signaling pathway (Wang et al., 2017; Hwarari et al., 2022). In this pathway, low temperature triggers plasma membrane rigidification and calcium influx into cells, which activates protein kinases, and in turn activates ICE1. Activated ICE1 triggers the expression of CBFs and other transcriptional regulators, which in turn regulate the expression of *COR* genes, and this results in cold tolerance (Wang et al., 2017; Liu et al., 2019). The ICE-CBF-COR pathway is associated with responses to cold stress in a wide variety of crop species (Wang et al., 2017; Hwarari et al., 2022). However, genes in the ICE-CBF-COR pathway have not been well characterized in chickpea. In our study, we found that the expression of key regulators such as *ICE1, COR47*, *COR314* and *LTI* were upregulated in Kesen_075 during cold acclimation while downregulated in CDC Consul. On the other hand*, CBF2* was upregulated during all freezing treatments in Kesen_075. Likewise, Akbari et al. (2023) detected increased expression of *COR47* at 4°C in cold tolerant and sensitive chickpea genotypes. Nevertheless, the increase in expression was more pronounced in the cold-tolerant genotype. *ICE1* further inhibits *MYB15*, which is a negative regulator (Agarwal et al., 2006). We found that *MYB15* is downregulated in Kesen_075 under cold acclimation while it was upregulated in CDC Consul. Our findings indicate that the ICE-CBF-COR pathway may play a significant role in chickpea’s cold tolerance.

Recent studies indicate that plant responses to cold stress are also regulated by various plant hormones either by CBF-dependent or -independent pathways (Shi et al., 2015; Raza et al., 2023). Most of the members of the *GA-2-oxidase* (*GA2oxs*) family, which encodes negative regulators of gibberellic acid and plant growth, are upregulated in Kesen_075 during all the treatments. Moreover, it has been found that the overexpression of *GA2oxs* can result in dwarf plants (Cheng et al., 2021). This may be one of the reasons that Kesen_075 is dwarfed in comparison to CDC Consul. AP2-like ABA repressor 1 (ABR1) is a repressor of abscisic acid and is induced by several stress conditions including cold (Pandey et al., 2005), whereas *ABA2* is an ABA biosynthesis gene and its overexpression causes enhanced tolerance to stress (Lin et al., 2007). In our study, one member of *ABR1* was upregulated while two members of *ABA2* were downregulated in CDC Consul, which could potentially contribute to the reduced cold stress tolerance observed in CDC Consul.

Signal transduction pathways are recognized for their pivotal role in responding to freezing stress. Notably, calcium ions (Ca^2+)^ serve as essential secondary messengers in plant systems (Yang et al., 2010). Genes associated with Ca^2+^ signaling pathways play indispensable roles in signaling linked to the freezing stress response (Niu et al., 2019). Our results indicate that genes encoding calcium dependent kinases, such as CIPK, CBL, CPK, CDPK, are upregulated in Kesen_075 during cold acclimation and recovery and this may indicate that these genes have an important role in cold tolerance in chickpea. In addition to Ca^2+^, ROS also play an important role in cold stress (Sun et al., 2019; Akbari et al., 2023). ROS serve as vital signaling molecules, facilitating plant responses to both abiotic and biotic stimuli, in addition to their involvement in various developmental processes (Huang et al., 2019; Dvořák et al., 2021). We found that the expression of genes encoding antioxidant enzymes was increased in both genotypes; however, genes encoding POD, TRX and GST were significantly increased in Kesen_075 during 3, 6, 12 and 24h of the freezing treatment, while no significant changes were detected in the expression of these genes in CDC Consul. This suggests that *POD*, *TRX* and *GST* may be key to the antioxidant response associated with cold tolerance in chickpea. ROS can also directly trigger the upregulation of genes involved in activating stress-related pathways, such as the MAPK cascade pathway (Zhou et al., 2020), which regulates plant resistance to temperature stress (Lin et al., 2021). In this study, we found that the expression of genes encoding MAP kinases increased in Kesen_075 during all the treatments, whereas they were downregulated in CDC Consul during cold acclimation.

Moreover, several key transcription factors, namely *NAC, bZIP, AP2/ERF, MYB, WRKY*, and *bHLH*, which have been previously reported to be associated with freezing stress (Shi et al., 2015; Zhu, 2016; Abdullah et al., 2022), exhibited significant upregulation in our study within the cold-tolerant genotype Kesen_075. Since transcription factors play a pivotal role in responding to cold stress by controlling the transcription of downstream genes that enhance a plant’s cold stress tolerance, they may serve as crucial regulators contributing to the cold tolerance observed in Kesen_075.

Additionally, we noticed the emergence of new roots in the wild accessions during recovery after subjecting them to freezing treatment. This phenomenon could potentially contribute to their heightened tolerance compared to the cultivars, which did not exhibit any signs of new root growth. Additionally, we observed anthocyanin pigmentation in the leaves and stems of the wild accessions, a trait that may be linked to their stress tolerance. In our study we have also found that genes related to anthocyanin biosynthesis, such as *C4H*, *CHS*, *anthocyanidin 3-O-glucosyltransferase* and *flavonol-3-O-glycoside-7-O-glucosyltransferase,* were upregulated in Kesen_075. This upregulation is likely responsible for the increased production of anthocyanin in this wild accession. Similar to our study, previous research has also demonstrated that anthocyanin accumulation can enhance plant tolerance to low temperatures (Zhang Q. et al., 2019; Li and Ahammed, 2023).

Using a QTL-seq bulked segregant analysis, we successfully identified genomic regions linked to freezing stress tolerance using an F2 population originating from a cross between CDC Consul and Kesen_075. We identified ten significant QTLs distributed across five chromosomes, all of which are associated with tolerance to freezing stress. Similarly, a recent study has also identified QTLs related to cold stress tolerance on Ch3 and Ch8 in chickpea (Mugabe et al., 2019). Intriguingly, genes that encode pivotal regulators of cold tolerance mechanisms, such as CBF2, important transcription factors like AP2/MYB/WRKY/bHLH/NAC/bZIP, as well as GA20ox (GA-20-oxidase), GA2ox, peroxidases, and protein kinases (Shi et al., 2015; Zhu, 2016; Lantzouni et al., 2020; Abdullah et al., 2022), were not only situated within the QTLs but also exhibited significant differential expression patterns, suggesting the importance of these genes in cold tolerance in chickpea.

## Conclusion

The present study explores the performance of cold-tolerant wild chickpea and cold-sensitive cultivated chickpea when exposed to freezing temperatures during an early vegetative growth stage. By utilizing transcriptome sequencing, we identified transcriptional responses to cold in both wild and cultivated chickpeas. We further identified candidate genes in wild chickpea that could be used for improving cold tolerance in cultivated chickpea. Important genes involved in signal transduction and transcriptional and posttranslational regulation exhibited different patterns of expression in wild and cultivated genotypes. Further, by selecting phenotypically extreme individuals from an F2 population resulting from a cross between wild and cultivated chickpea genotypes and conducting a QTL-seq analysis, we were able to identify QTLs linked to resistance against freezing stress. Furthermore, we identified candidate genes within these QTL regions that have previously been reported to play pivotal roles in mechanisms related to cold stress tolerance.

In conclusion, our study provides a comprehensive understanding of chickpea’s response to freezing stress, shedding light on the roles of wild progenitors, differential gene expression patterns, functional annotations, and QTLs associated with cold tolerance. These findings pave the way for targeted breeding strategies aimed at enhancing chickpea’s cold stress resilience, thus bolstering global food security.

## Data availability statement

The datasets presented in this study can be found in online repositories. The names of the repository/repositories and accession number(s) can be found below: BioProject ID PRJNA1034600.

## Author contributions

SK: Data curation, Formal Analysis, Investigation, Methodology, Writing – original draft. MH: Data curation, Formal Analysis, Investigation, Methodology, Writing – review & editing. BT: Conceptualization, Investigation, Project administration, Resources, Supervision, Writing – review & editing.

## Supplementary material

Supplementary Figure 1 Cold tolerance ratings of selected *C. reticulatum* wild accessions and chickpea cultivars (CDC Consul and CDC Leader) following recovery after 24hr of freezing conditions at -6°C (n=3) with cold acclimation (blue bars) and without cold acclimation (orange bars). Data are presented as mean ± SD.

Supplementary Figure 2 Library sizes of all RNA-seq samples.

Supplementary Figure 3 Density of log-CPM (log counts per million) values before **(A)** and after **(B)** filtering out genes with low read counts.

Supplementary Figure 4 Gene ontology enrichment test results of all differentially expressed genes in **(A)** cold sensitive cultivar CDC Consul and **(B)** cold tolerant wild accession Kesen_075.

Supplementary Figure 5 Responses of one **(A)** cold tolerant and **(B)** one sensitive F2 line from the population of 197 F2 lines derived from the cross between cold tolerant wild accession Kesen_075 and cold sensitive cultivar CDC Consul after one week of recovery following 24 hours of freezing treatment at -6°C.
