# Supplementary figures and images for: Freezing stress response of wild and cultivated chickpeas
Source: Front Plant Sci. 2024 Feb 5;14:1310459. doi: 10.3389/fpls.2023.1310459 (PMC10876003; doi:10.3389/fpls.2023.1310459)

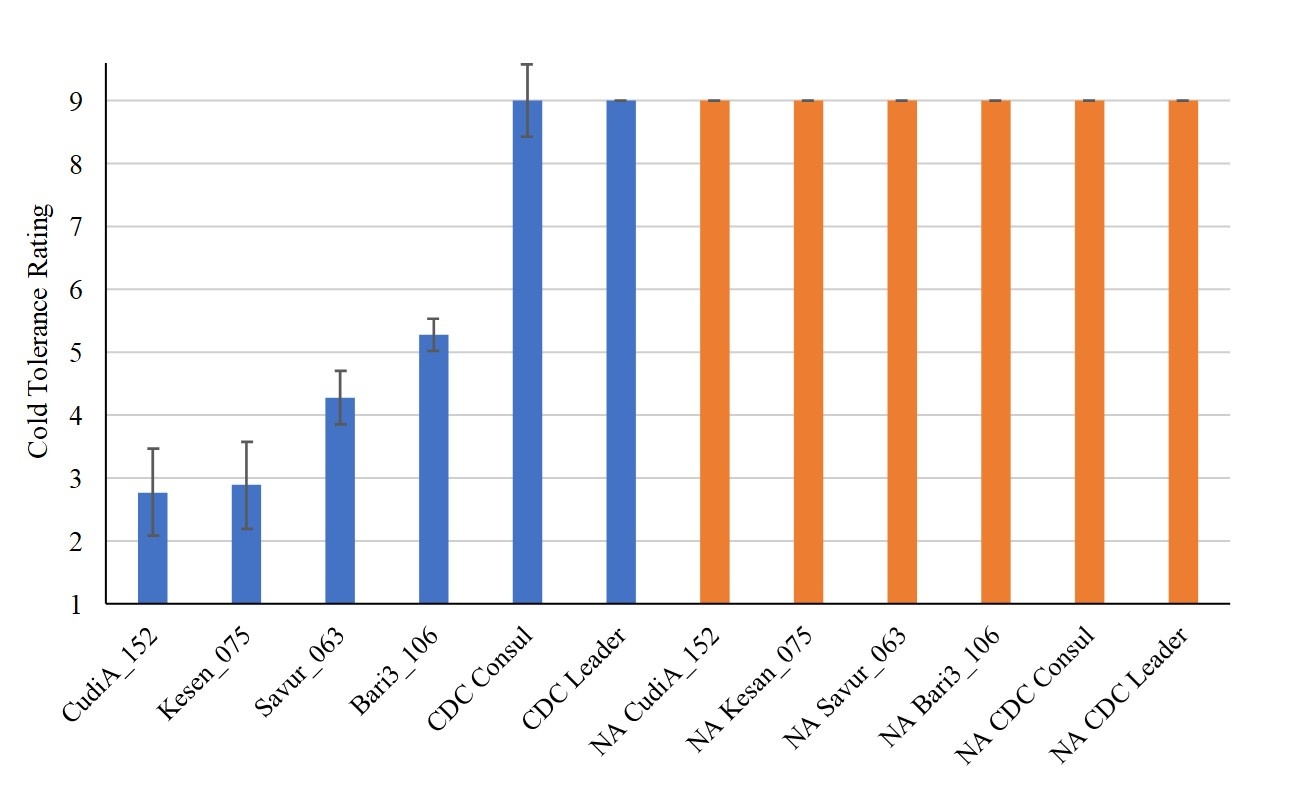

Supplement: Supplementary Figure 1 — Cold tolerance ratings of selected C. reticulatum wild accessions and chickpea cultivars (CDC Consul and CDC Leader) following recovery after 24hr of freezing conditions at -6°C (n=3) with cold acclimation (blue bars) and without cold acclimation (orange bars). Data are presented as mean ± SD. [file Image_1.jpeg]

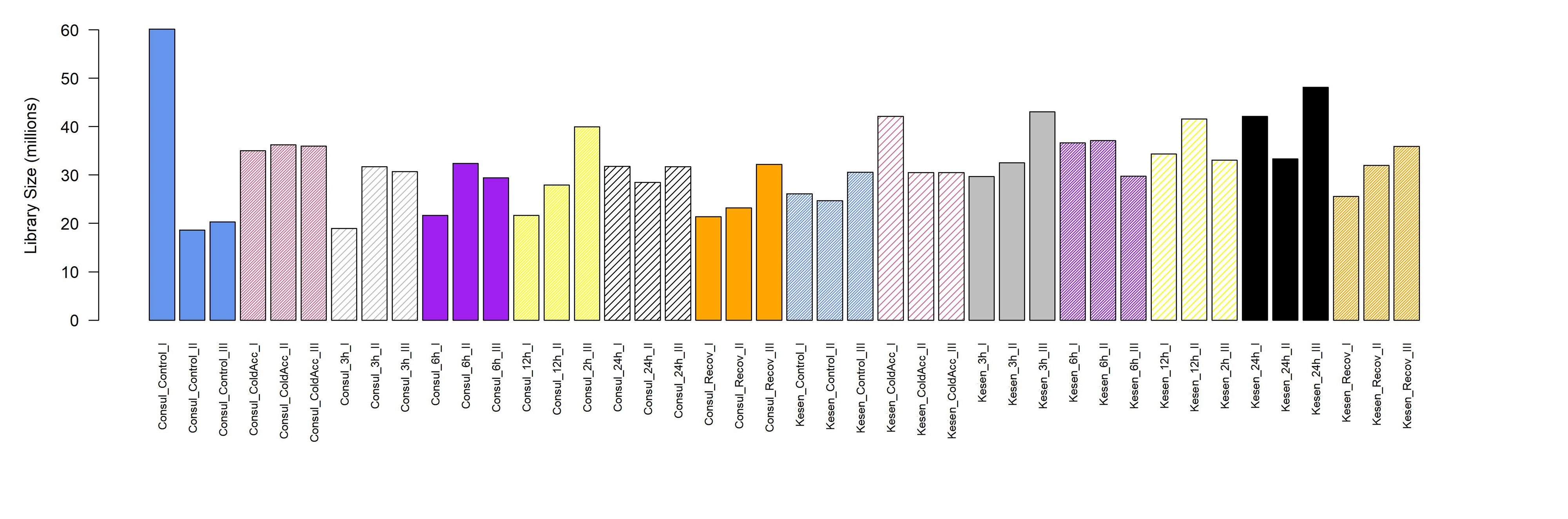

Supplement: Supplementary Figure 2 — Library sizes of all RNA-seq samples. [file Image_2.jpeg]

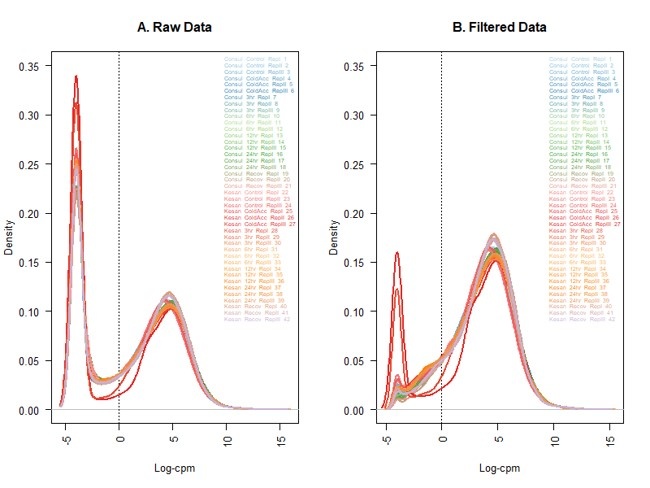

Supplement: Supplementary Figure 3 — Density of log-CPM (log counts per million) values before (A) and after (B) filtering out genes with low read counts. [file Image_3.jpeg]

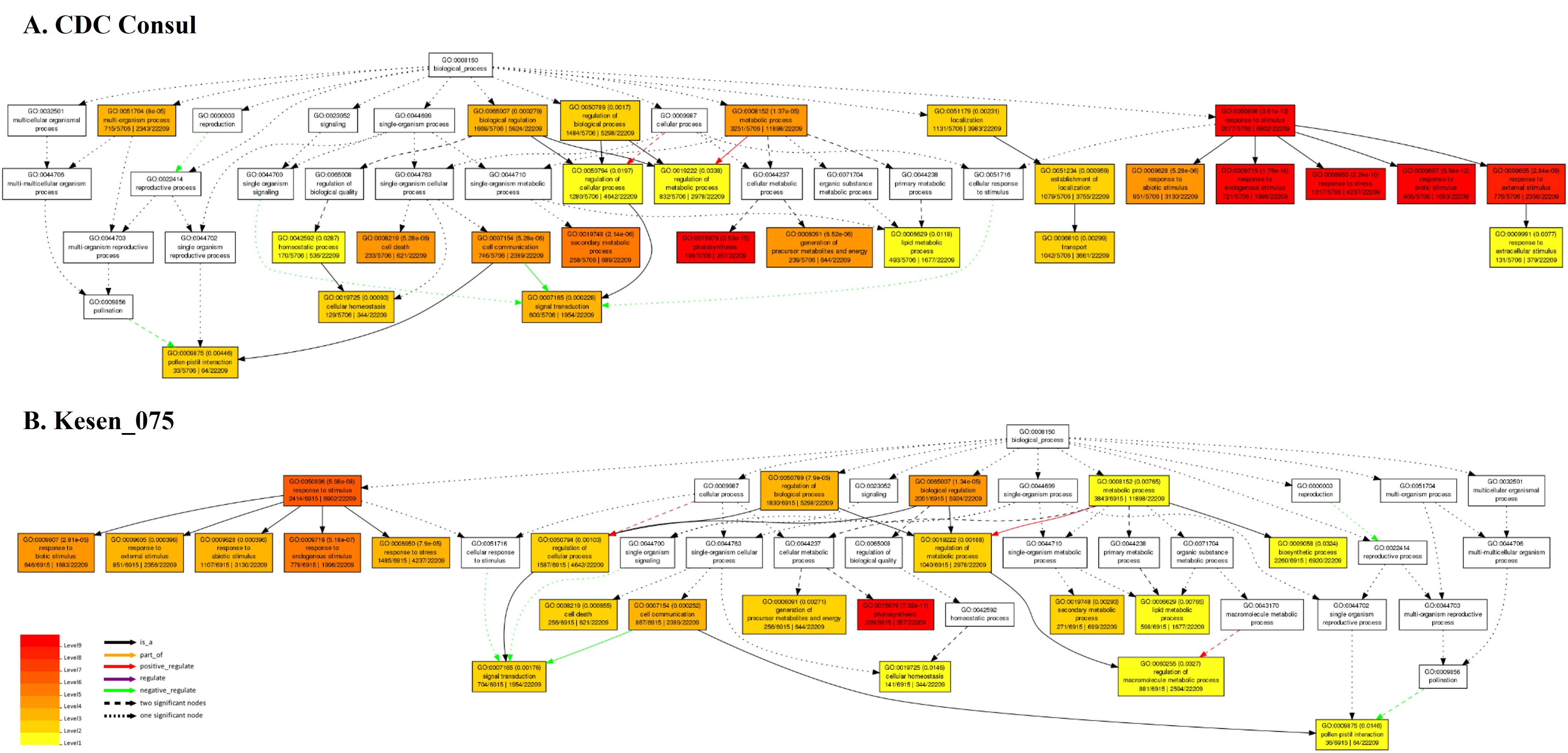

Supplement: Supplementary Figure 4 — Gene ontology enrichment test results of all differentially expressed genes in (A) cold sensitive cultivar CDC Consul and (B) cold tolerant wild accession Kesen_075. [file Image_4.jpeg]

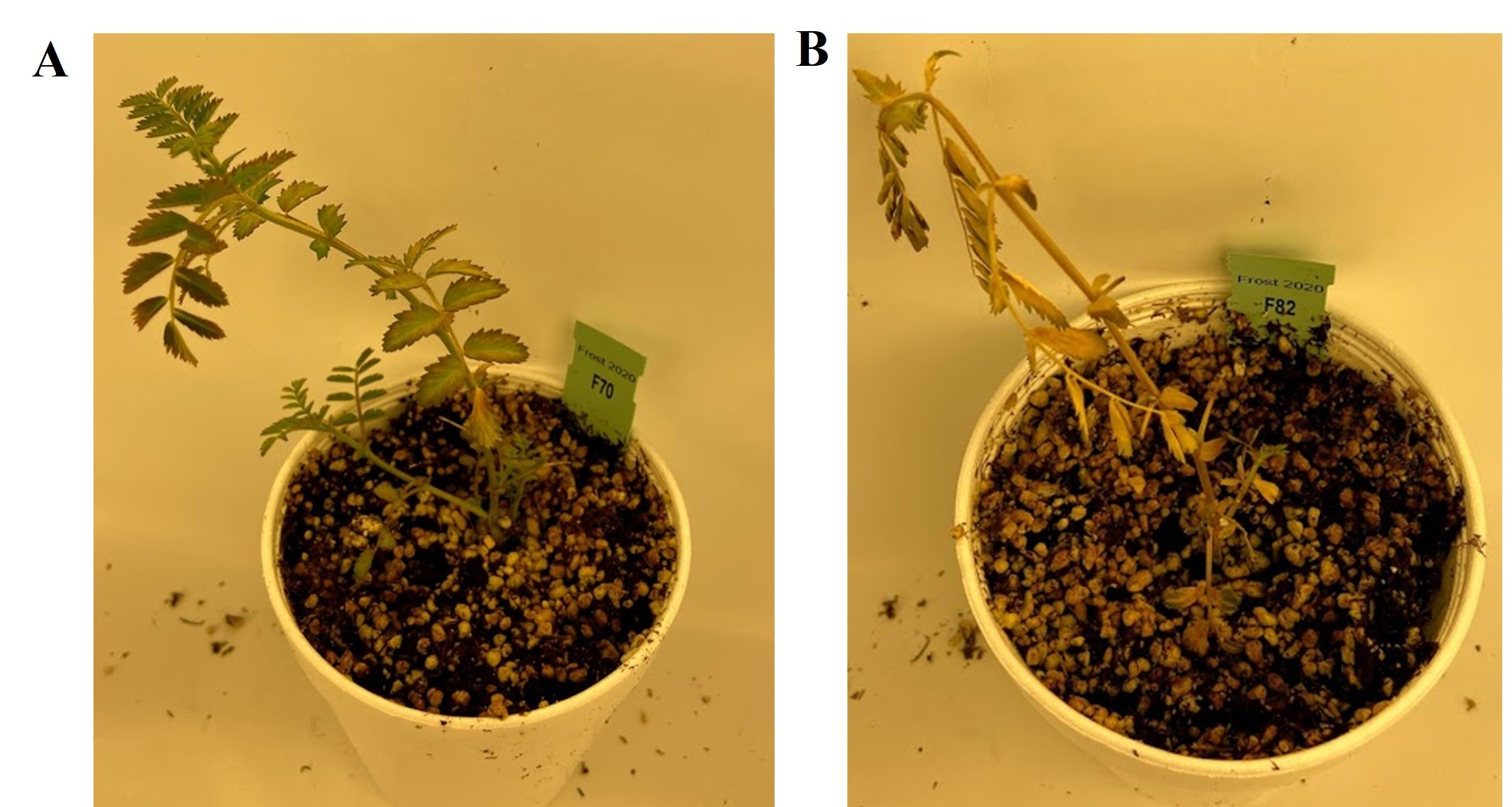

Supplement: Supplementary Figure 5 — Responses of one (A) cold tolerant and (B) one sensitive F2 line from the population of 197 F2 lines derived from the cross between cold tolerant wild accession Kesen_075 and cold sensitive cultivar CDC Consul after one week of recovery following 24 hours of freezing treatment at -6°C. [file Image_5.jpeg]
