# Supplementary material for: Freezing stress response of wild and cultivated chickpeas
Source: Front Plant Sci. 2024 Feb 5;14:1310459. doi: 10.3389/fpls.2023.1310459 (PMC10876003; doi:10.3389/fpls.2023.1310459)
Supplement: Supplementary file 6 [file Table_1.docx]

|  |  | **# of Reads Sequenced** | **# of Read-Pairs After Trimming** | **% Uniquely Mapped** | **Mapped Read-Pair Length** | **% Mapped to Multiple Loci** |
| --- | --- | --- | --- | --- | --- | --- |
| Consul | Control | 78327197 | 78323067 | 93% | 296.6 | 3% |
|  | Cold Acclimation | 85744893 | 85739741 | 94% | 296.6 | 3% |
|  | 3 hr frost | 65230837 | 65226690 | 93% | 296.7 | 3% |
|  | 6 hr frost | 66696088 | 66692224 | 93% | 296.7 | 3% |
|  | 12 hr frost | 71764499 | 71760251 | 93% | 296.7 | 3% |
|  | 24 hr frost | 74045962 | 74041597 | 93% | 296.6 | 3% |
|  | Recovery | 61317493 | 61313823 | 93% | 296.6 | 3% |
| **Average** |  | **71875281.3** | **71871056** | **93.14%** | **296.64** | **3%** |
| Kesen_075 | Control | 65515822 | 65512808 | 92% | 295.9 | 4% |
|  | Cold Acclimation | 84667804 | 84661417 | 91% | 295.8 | 4% |
|  | 3 hr frost | 86743302 | 86737289 | 91% | 295.7 | 4% |
|  | 6 hr frost | 84674185 | 84669361 | 92% | 295.9 | 3% |
|  | 12 hr frost | 90924835 | 88572715 | 92% | 295.9 | 3% |
|  | 24 hr frost | 100313147 | 100307351 | 92% | 295.8 | 4% |
|  | Recovery | 89995283 | 89990368 | 80% | 296.1 | 3% |
| **Average** |  | **86119196.9** | **85778758** | **90%** | **295.87** | **3.57%** |

**Supplemental Table 1.** Percentages of paired-end reads mapped to the chickpea reference genome.
